# High-Purity CTC RNA Sequencing Identifies Prostate Cancer Lineage Phenotypes Prognostic for Clinical Outcomes

**DOI:** 10.1158/2159-8290.CD-24-1509

**Published:** 2025-02-06

**Authors:** Marina N. Sharifi, Jamie M. Sperger, Amy K. Taylor, Katharine E. Tippins, Shannon R. Reese, Viridiana Carreno, Katherine R. Kaufmann, Alex H. Chang, Luke A. Nunamaker, Charlotte Linebarger, Leilani Mora-Rodriguez, Jennifer L. Schehr, Hannah M. Krause, Kyle T. Helzer, Matthew L. Bootsma, Grace C. Blitzer, John M. Floberg, Christos E. Kyriakopoulos, Hamid Emamekhoo, Elisabeth I. Heath, Meghan Wells, Scott T. Tagawa, Martin Sjöström, Atish D. Choudhury, Menggang Yu, Andrew J. Armstrong, Dana E. Rathkopf, Himisha Beltran, Peter S. Nelson, Felix Y. Feng, Scott M. Dehm, David Kosoff, Xiao X. Wei, Rana R. McKay, Shuang G. Zhao, Joshua M. Lang

**Affiliations:** 1 Carbone Cancer Center, University of Wisconsin–Madison, Madison, Wisconsin.; 2 Department of Medicine, University of Wisconsin–Madison, Madison, Wisconsin.; 3 Department of Human Oncology, University of Wisconsin–Madison, Madison, Wisconsin.; 4 Karmanos Cancer Institute, Wayne State University, Detroit, Michigan.; 5 Weill Cornell Medical College, New York, New York.; 6 Helen Diller Family Comprehensive Cancer Center, University of California, San Francisco, San Francisco, California.; 7 Department of Radiation Oncology, University of California, San Francisco, San Francisco, California.; 8 Division of Oncology, Department of Clinical Sciences Lund, Lund University, Lund, Sweden.; 9 Department of Hematology, Oncology and Radiation Physics, Skåne University Hospital, Lund, Sweden.; 10 Dana-Farber Cancer Institute, Boston, Massachusetts.; 11 Department of Biostatistics, University of Michigan School of Public Health, Ann Arbor, Michigan.; 12 Duke Cancer Institute Center for Prostate and Urologic Cancer, Duke University, Durham, North Carolina.; 13 Division of Solid Tumor Oncology, Department of Medicine, Genitourinary Oncology Service, Memorial Sloan Kettering Cancer Center, New York, New York.; 14 Division of Human Biology, Fred Hutchinson Cancer Center, Seattle, Washington.; 15 Department of Urology, University of Washington, Seattle, Washington.; 16 Masonic Cancer Center, University of Minnesota, Minneapolis, Minnesota.; 17 Department of Laboratory Medicine and Pathology, University of Minnesota, Minneapolis, Minnesota.; 18 Department of Urology, University of Minnesota, Minneapolis, Minnesota.; 19 William S. Middleton Memorial Veterans’ Hospital, Madison, Wisconsin.; 20 Moores Cancer Center, University of California San Diego, La Jolla, California.

## Abstract

The development of treatment resistance remains universal for patients with metastatic prostate cancer, driven by androgen receptor alterations and lineage state transitions. Identifying the evolution of lineage transitions in treatment resistance has been limited by the challenges of collecting serial tissue biopsies on treatment, which can be overcome using blood-based liquid biopsies. Using a novel circulating tumor cell (CTC) isolation approach, we collected 273 CTC samples from 117 patients with metastatic prostate cancer for RNA sequencing. One hundred forty-six samples from 70 patients had tumor purity comparable with tissue biopsies. We identified four CTC transcriptional phenotypes, mirroring lineage states identified in the tissue. Patients with a luminal-B–like CTC phenotype defined by persistent androgen receptor signaling and high proliferation, as well as those with a neuroendocrine CTC phenotype, had significantly shorter survival than patients with luminal-A–like and low proliferation phenotypes. In a prospective substudy, pretreatment CTC luminal-B–like phenotype was associated with early progression on ^177^Lu–PSMA-617.

**Significance:**

Treatment resistance remains a universal driver of lethal metastatic prostate cancer, associated with acquired genomic alterations and lineage transitions. Using a novel high-purity CTC isolation approach for CTC transcriptional profiling, we identified four lineage phenotypes differentially associated with prognosis in metastatic prostate cancer.

## Teaser

Circulating tumor cell (CTC) lineage phenotypes are associated with prognosis in metastatic prostate cancer, and the CTC luminal-B-like phenotype is associated with early progression on 177Lu-PSMA-617.

## Introduction

The greatest improvements in survival for patients with metastatic castration-sensitive prostate cancer (mCSPC) have been found with the addition of androgen receptor pathway inhibitors (ARPI) with or without chemotherapy to androgen deprivation therapy for newly diagnosed disease. However, the eventual development of castration-resistant prostate cancer (mCRPC) remains nearly universal. The mechanisms that drive resistance to androgen receptor (AR)–directed therapies are varied and can include acquired AR genomic and transcriptomic alterations that drive pathway activation (1–4) and lineage state transitions that bypass AR signaling and culminate in a small-cell/neuroendocrine prostate cancer (NEPC) phenotype (5–8). These lineage states are defined by transcriptional and epigenetic phenotypes and extend beyond AR-driven adenocarcinomas and NEPC (9–13). After treatment with AR-directed therapies, there are few treatment options that improve survival, limited to taxane chemotherapy, PARP inhibitors for genomically selected patients, and more recently, lutetium-177–PSMA-617 (^177^Lu–PSMA-617). ^177^Lu–PSMA-617 is a radioligand therapy targeting prostate-specific membrane antigen (PSMA), a highly expressed protein on the surface of prostate adenocarcinoma cells (14). It was FDA-approved in 2022 after demonstrating improved survival in patients with taxane-pretreated PSMA–PET–positive mCRPC in the phase III VISION trial (15). However, although 85% of screened patients on this trial had PSMA–PET–positive disease, more than half of these patients had intrinsic resistance to ^177^Lu–PSMA-617, and acquired resistance is common, highlighting the need to better define mechanisms of treatment response and resistance with this therapy. The contribution of lineage state transitions to ^177^Lu–PSMA-617 resistance is not well understood.

Identifying distinct lineage transitions that drive treatment resistance in prostate cancer has been limited by the challenges of performing serial tumor biopsies for transcriptional phenotyping in sufficiently large numbers of patients. Blood-based “liquid” biopsies more easily enable the longitudinal molecular analysis that is required to fully understand the evolutionary dynamics of treatment resistance, especially in contemporary cohorts treated with new therapies such as ^177^Lu–PSMA-617. Early liquid biopsy studies identified circulating tumor cell (CTC) enumeration and expression of the *AR-V7* splice variant as biomarkers of resistance to ARPIs (16, 17). Cell-free DNA (cfDNA) sequencing is the most widely used clinical liquid biopsy platform and has identified circulating tumor DNA content and AR genomic alterations as biomarkers that associate with ARPI resistance and poor survival (18). In addition to sequence variant analysis, studies of cfDNA methylation and fragmentation patterns can identify a subset of patients with NEPC (6, 19–22). CTC gene expression profiling with tumor-specific qRT-PCR panels have also been prognostic for response to ARPIs (16, 23, 24) and can detect the transition to NEPC (25).

Powerful as these methods are, they do not provide the complete transcriptional analysis of complex gene expression signatures important in lineage plasticity that have been defined in solid tumor studies (5, 7, 9, 10, 26). Reliable evaluation of gene expression in CTCs would represent a liquid biopsy that can match the capabilities of tissue-based gene expression profiling. However, it is challenging to capture sufficiently high numbers of CTCs with tumor purity levels mirroring tissue studies. These issues are critical in the study of lineage state transitions as many of the transcripts in these signatures are not tumor specific (27). We have therefore developed a novel approach to CTC purification and RNA sequencing (RNA-seq) that results in purity comparable with tumor biopsies. We used this high-purity CTC method to perform the first large-scale CTC RNA-seq study in 273 blood samples from a multi-institutional cohort of 117 patients with metastatic prostate cancer. We identify distinct prostate cancer lineage states previously described only via tissue profiling studies that are associated with prognosis in our cohort. These distinct lineage states also exhibit differential expression of cell-surface targets with agents under investigation in prostate cancer. Finally, we demonstrate the evolution of lineage states on-treatment, including the investigation of lineage states during treatment with ^177^Lu–PSMA-617.

## Results

### Novel CTC Isolation for High-Purity RNA-seq

**Figure 1.**
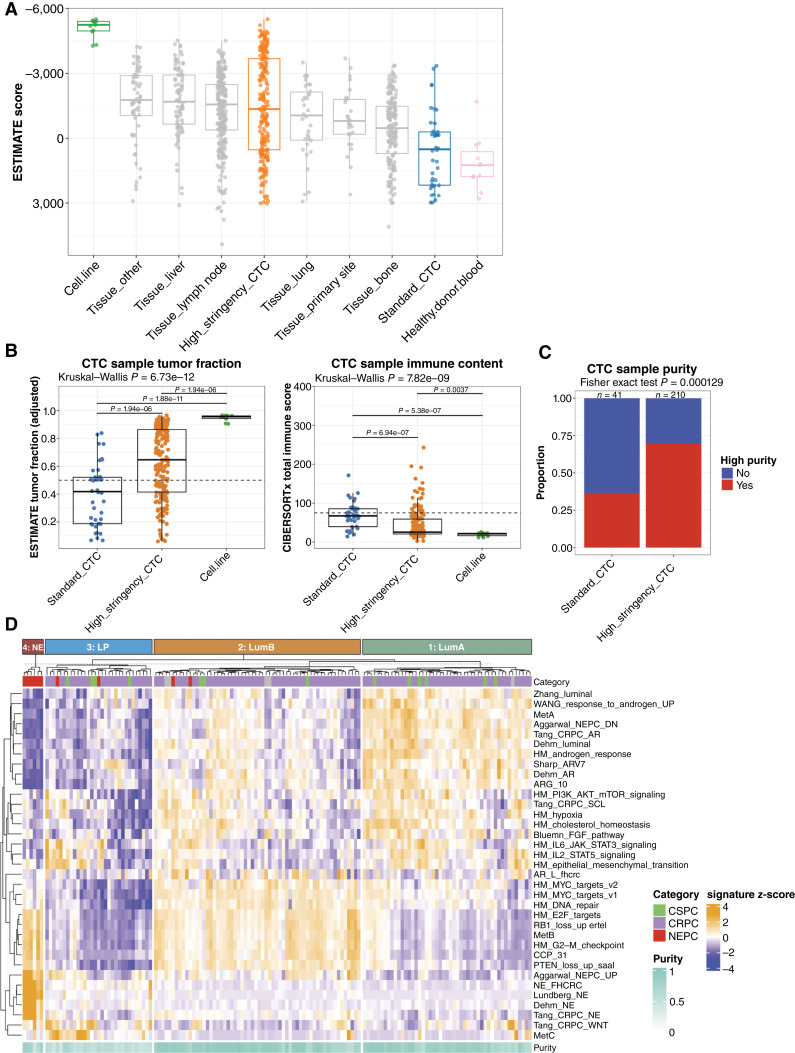
RNA-seq of high-purity CTCs isolated from patients with metastatic prostate cancer reveals four transcriptional phenotypes. **A,** Tumor/CTC purity was assessed using the ESTIMATE algorithm in prostate cancer cell line samples (*n* = 12, green), CTC samples processed with standard-stringency (*n* = 41, blue) or novel high-stringency (*n* = 211, orange) method; healthy donor blood samples processed analogous to novel high-stringency CTC samples (*n* = 12, pink) and a published dataset of mCRPC tissue biopsies separated by biopsy site (*n* = 629, gray). ESTIMATE score is plotted for each sample. **B,** ESTIMATE tumor fraction (left) and CIBERSORTx immune content (right) scores for standard-stringency (*n* = 41, blue) or high-stringency (*n* = 211, orange) CTC methods are plotted and compared with prostate cancer cell lines (*n* = 12, green). **C,** Assessment of the number of samples meeting high-purity threshold for standard-stringency vs. high-stringency methods. **D,** Consensus *k*-means clustering of single-sample pathway *z*-scores of 146 high-purity CTC samples from 70 patients with metastatic prostate cancer identifies four distinct clusters. CRPC, castration-resistant prostate cancer.

We previously developed an automated platform for high-throughput CTC capture and RNA extraction that leverages sequential negative immune cell selection followed by positive selection of CTCs to improve CTC recovery for targeted transcriptional profiling (i.e., “standard” method; ref. 28). To adapt this approach for bulk CTC RNA-seq, which requires higher tumor purity, we integrated a higher stringency negative selection approach with this platform. To infer CTC sample tumor purity, we used a slight modification of the ESTIMATE algorithm (see “Methods”; Supplementary Fig. S1A–S1C; ref. 29). We compared ESTIMATE scores from high-stringency and standard methods with cell lines, healthy donor blood samples, and a published dataset of 634 mCRPC tissue biopsies annotated with the biopsy site (11). We observed more negative ESTIMATE scores, indicative of higher tumor purity, for CTC samples isolated with the high- versus standard-stringency approach. In contrast to the standard-stringency CTC samples, ESTIMATE scores for the high-stringency CTC samples were comparable with those of tissue biopsies (Fig. 1A). As an orthogonal approach to the estimated tumor fraction from the ESTIMATE algorithm, CIBERSORTx was used to infer immune content because the predominant benign tissue background in CTC samples is white blood cells (30). Inferred tumor purity was significantly higher in CTC samples processed with our new high-stringency approach (*P* < 0.0001), whereas the CIBERSORTx absolute immune content scores were significantly lower (*P* < 0.0001; Fig. 1B). To identify CTC samples with sufficient tumor purity for downstream global transcriptional pathway analysis, we used a conservative combined threshold incorporating tumor purity, epithelial gene expression, and CIBERSORTx immune content assessment (see “Methods”). Notably, although less than half of the metastatic prostate cancer CTC samples isolated with the standard-stringency condition met this threshold, 70% of sequenced samples isolated with the high-stringency approach met this threshold (Fig. 1C), indicating the broad potential applicability of this platform for CTC transcriptional profiling in patients with metastatic prostate cancer.

### Multi-institutional CTC RNA-seq Dataset

Two hundred seventy-three longitudinal blood samples from 117 patients with metastatic prostate cancer from the University of Wisconsin, University of California San Diego, and the William S. Middleton Memorial Veterans Administration Hospital (Madison, Wisconsin) were processed for high-stringency CTC isolation, RNA extraction, and RNA-seq. At the time of first CTC collection, 17% of patients had mCSPC, 80% had mCRPC, and 3% had biopsy-proven NEPC; 61% had prior ARPI therapy; and 49% were diagnosed with *de novo* metastatic disease (Table 1). The median serum PSA was 30.1 ng/mL. Fifty-seven percent of patients had lymph node involvement, 80% had bone involvement, 12% had non-liver visceral involvement, and 17% had liver involvement (Table 1).

**Table 1.** Clinical characteristics of cohort and CTC phenotype groups.

Characteristic	Total cohort	Low_CTC	LP	LumA	LumB	NE
Number of patients	117	63	12	21	18	3
Age, median (range), years	71 (53–96)	70 (57–96)	71 (56–83)	73 (53–84)	72 (57–88)	65 (64–72)
PSA at blood draw median (range), ng/mL	30.1 (0–3,334)	14.3 (0–290.4)	23.9 (0.9–816.4)	110.3 (10.5–1,896.3)	63.5 (0.1–3,334)	0.32 (0.1–1.1)
Gleason score, *n* (%)						
≤6	5 (4)	3 (5)	1 (8)	0 (0)	1 (5)	0 (0)
7	16 (14)	10 (16)	2 (17)	1 (5)	3 (17)	0 (0)
8	15 (13)	11 (17)	1 (8)	2 (10)	1 (5)	0 (0)
≥9	45 (38)	23 (36)	5 (42)	8 (38)	6 (33)	3 (100)
Unknown	36 (31)	16 (25)	3 (25)	10 (48)	7 (39)	0 (0)
*De novo* metastatic disease, *n* (%)						
Yes	57 (49)	29 (46)	5 (42)	6 (29)	10 (55)	3 (100)
No	40 (34)	23 (36)	6 (50)	10 (48)	5 (28)	0 (0)
Unknown	20 (17)	11 (17)	1 (8)	5 (24)	3 (17)	0 (0)
Metastatic sites, *n* (%)						
Lymph node	67 (57)	37 (59)	9 (75)	10 (48)	9 (50)	2 (67)
Bone	94 (80)	48 (76)	10 (83)	18 (86)	15 (83)	3 (100)
Non-liver visceral	14 (12)	7 (11)	3 (25)	2 (10)	1 (4)	1 (33)
Liver	20 (17)	5 (8)	3 (25)	2 (10)	10 (56)	1 (33)
Unknown	12 (10)	8 (13)	1 (8)	3 (14)	0 (0)	0 (0)
Type of disease, *n* (%)						
CSPC	20 (17)	14 (22)	0 (0)	5 (24)	1 (6)	0 (0)
CRPC	93 (80)	49 (78)	11 (92)	16 (76)	17 (94)	0 (0)
NEPC	4 (3)	(0)	1 (8)	0 (0)	0 (0)	3 (1)
Months since diagnosis median (range)	44.7 (1.1–250)	43.4 (1.4–221)	94.5 (11.5–143)	59.7 (1.1–250)	42.5 (6.0–245)	29.7 (8.0–57.4)
Months on current therapy median (range)	3.0 (0–92.3)	2.7 (0–92.3)	4.1 (0–10.0)	4.2 (0–11.9)	3.5 (0–8.5)	0 (0–0.9)
Prior treatment, *n* (%)						
ARPI	72 (61)	34 (54)	10 (83)	13 (62)	13 (72)	2 (67)
Chemotherapy	46 (39)	21 (33)	7 (58)	9 (43)	8 (44)	1 (33)
Radium-223	8 (7)	4 (6)	1 (8)	2 (9)	1 (5)	0 (0)
^177^Lu–PSMA-617	6 (5)	2 (3)	1 (8)	1 (5)	2 (10)	0 (0)
PARP inhibitor	6 (5)	4 (6)	1 (8)	1 (5)	0 (0)	0 (0)

Abbreviation: CRPC, castration-resistant prostate cancer.

### CTC RNA-seq Reveals Transcriptional Phenotypes Mirroring Tissue and Associated with Prognosis

Two hundred ten samples from 99 patients recovered adequate RNA for sequencing. One hundred forty-six samples from 70 patients met the high tumor purity threshold for single-sample pathway analysis, which was performed for published gene sets associated with prostate cancer transcriptional phenotypes, androgen signaling, luminal identity, neuroendocrine differentiation, and treatment resistance (4, 5, 9, 10, 26, 31–36), as well as Molecular Signatures Database hallmark pathway gene sets associated with proliferation, growth factor signaling, and metabolism (Supplementary Material S1; ref. 37). RNA-seq quality metrics suggest that the low-purity samples may contain fewer cells than the high-purity samples (Supplementary Fig. S2); cDNA yield was lower, and although the number of total and uniquely mapped sequences recovered were similar, the number of unique genes detected were lower. Immune content, ESTIMATE scores, and predicted tumor fraction were highly reproducible in nine replicate CTC samples, including three sets of low-purity and six sets of high-purity samples (Supplementary Fig. S3A). The expression of individual genes and single sample pathway activity scores discussed below were highly reproducible in samples with high purity, whereas more variable in the low-purity samples (Supplementary Fig. S3B and S3C).

**Figure 2.**
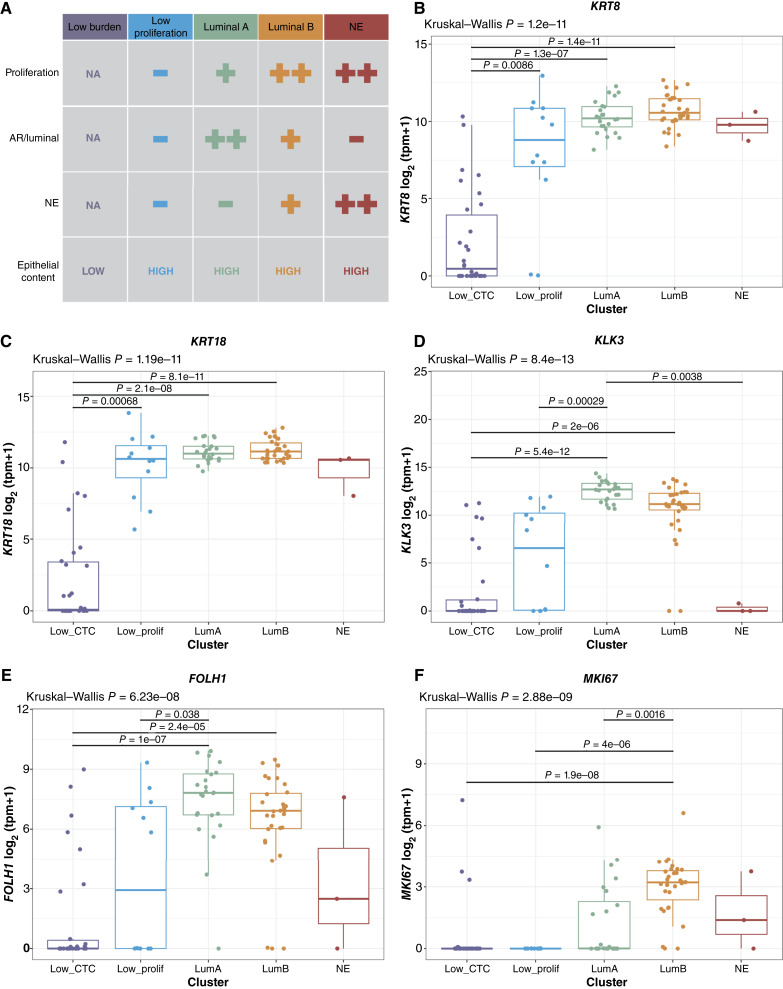
Gene expression of epithelial and prostate adenocarcinoma–associated genes across CTC phenotypes. **A,** Schematic of the CTC transcriptional phenotypes. **B** and **C,** Epithelial keratin (*KRT8* and *KRT18*) gene expression across phenotypes; for patients with multiple CTC samples, the highest purity sample is included (Low_CTC, *n* = 30; Low_prolif, *n* = 12; LumA, *n* = 24; LumB, *n* = 31; and NE, *n* = 3). **D,***KLK3* (PSA), (**E**) *FOLH1* (PSMA), and (**F**) *MKI67* (Ki-67) gene expression across phenotypes; for patients with multiple CTC samples, the highest purity sample is included (Low_CTC, *n* = 30; Low_prolif, *n* = 12; LumA, *n* = 24; LumB, *n* = 31; and NE, *n* = 3).

Consensus k-means clustering of the pathway scores identified four clusters (Fig. 1D). Clusters 1 and 2 had high AR signaling/luminal pathway scores and low neuroendocrine scores but were distinguished by lower (cluster 1) versus higher (cluster 2) proliferation scores, analogous to the luminal-A and luminal-B transcriptional phenotypes first described in breast cancer (38, 39) and more recently in prostate cancer (11–13, 40, 41). Cluster 3 consisted of patients with low AR/luminal, low neuroendocrine, and low proliferation scores. Cluster 4 had low AR/luminal scores, high proliferation scores, and high NEPC scores and consisted entirely of CTCs isolated from patients with histologically confirmed NEPC. Based on these clusters, we classified each sample in our cohort into one of five CTC phenotypes: (i) low CTC burden (not meeting sample tumor purity threshold for gene expression analysis), (ii) cluster 1/luminal-A–like (LumA), (iii) cluster 2/luminal-B–like (LumB), (iv) cluster 3/low proliferation (LP), and (v) cluster 4/neuroendocrine (NE; Fig. 2A).

There was no difference in sample processing time or shipped versus non-shipped status across the CTC phenotypes (Supplementary Fig. S4), indicating that these key preanalytic technical variables are not confounding factors. Tumor fraction was highest in the LumA/B samples, moderately lower in the LP and NE samples, and lowest, as expected, in the low CTC burden samples (Supplementary Fig. S5A and S5B). Conversely, immune content was lowest in the LumA/B and NE samples, modestly higher in the LP samples, and highest in the low CTC burden samples (Supplementary Fig. S5C and S5D). Because the LP samples, despite meeting tumor purity and immune content thresholds for pathway analysis, were low in both AR/luminal and neuroendocrine pathway scores and had lower tumor fraction than the LumA/B samples (although similar to the NE samples), we evaluated epithelial keratin gene expression across the CTC phenotypes as an independent measure of epithelial/CTC content. In contrast to the low CTC samples that did not meet the purity threshold for gene expression analysis, samples from all four high-purity clusters including the LP cluster had similar epithelial keratin expression consistent with similar absolute epithelial/tumor cell content (Fig. 2B and C; Supplementary Fig. S6A and S6B). *KLK3* (PSA) and *FOLH1* (PSMA) were most highly expressed in the LumA and LumB samples but were also expressed at a lower level in the LP samples and a subset of the low CTC burden samples, confirming the identity of the epithelial portion of these samples as prostate CTCs (Fig. 2D and E; Supplementary Fig. S6C and S6D). As expected, the NE samples did not express *KLK3* or *FOLH1* but did demonstrate expression of neuroendocrine genes including *ASCL1*, *INSM1*, and *SYP* (Supplementary Fig. S7A–S7F). Finally, *MKI67* (Ki-67) expression was high in the LumB and NE samples, lower in the LumA samples, and essentially absent in the low-proliferation samples (Fig. 2F; Supplementary Fig. S8), concordant with the proliferation pathway scores (Fig. 1D). Ki-67 protein expression has also been described in CTCs (42, 43), and because the survival of CTCs in circulation is quite short, likely less than 3 hours (44), proliferative features such as those seen in the LumB and NE CTC samples may simply reflect the proliferative capacity/state of the tumor from which they were shed.

**Figure 3.**
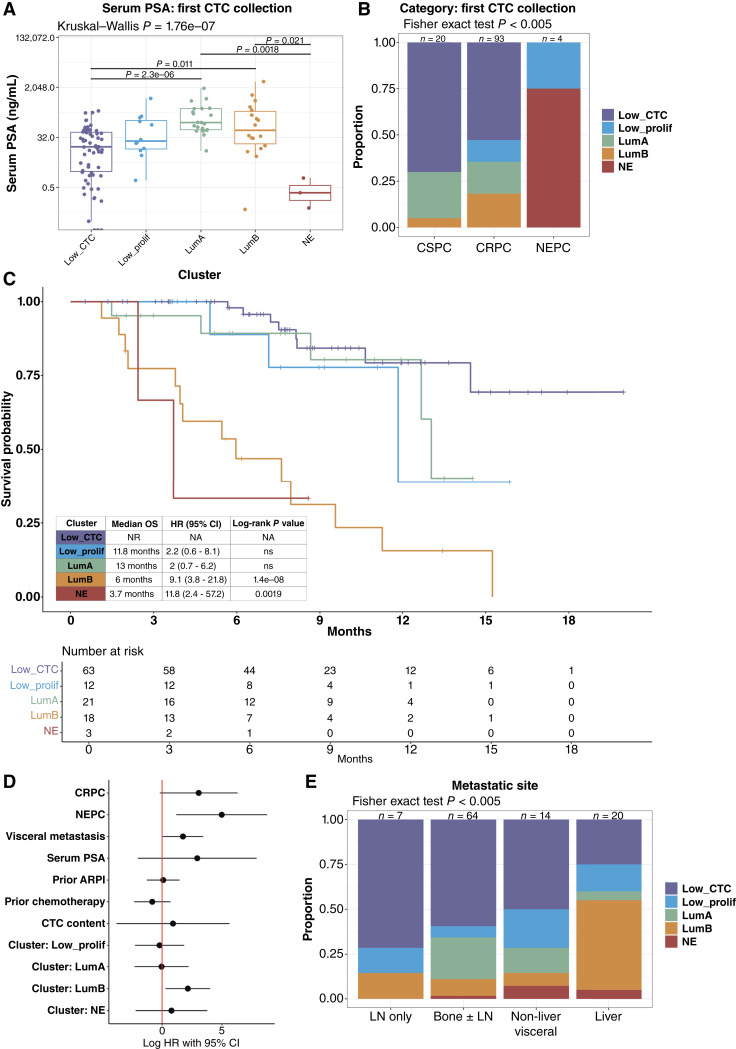
Luminal-B CTC phenotype is associated with poor prognosis and adverse clinical features. **A,** Serum PSA (ng/mL) at the time of CTC collection in the survival analysis subset (Low_CTC, *n* = 63; Low_prolif, *n* = 12; LumA, *n* = 21; LumB, *n* = 18; and NE, *n* = 3). **B,** Proportion of samples in each CTC transcriptional phenotype for each disease category in the survival analysis subset (mCSPC, *n* = 20; mCRPC, *n* = 93; and NEPC, *n* = 4) is shown. **C,** Kaplan–Meier plot of the OS from the time of the first CTC collection for each patient (*n* = 117) by CTC transcriptional phenotype at the first CTC collection. Median survival, and HR and log-rank *P* value relative to low CTC burden (Low_CTC) phenotype are shown in the inset. Risk table is shown below. **D,** Multivariate analysis of the CTC phenotype clustersgroups demonstrates that LumB remains a poor prognostic factor after adjusting for adverse clinical features. **E,** Distribution of CTC transcriptional phenotypes by metastatic sites involved at the time of the first CTC collection including lymph node only (LN only, *n* = 7), bone ± lymph node (bone ± LN, *n* = 64), at least one non-liver soft-tissue site (non-liver visceral, *n* = 14), and at least one liver site (liver, *n* = 20).

Clinical characteristics across the CTC phenotype groups are summarized in Table 1. Serum PSA was highest in the LumA and LumB samples, lower in the LP and low_CTC samples, and lowest in NE samples (Fig. 3A; Table 1; Supplementary Fig. S9A). Samples from patients with mCSPC had a higher proportion of low-CTC and lower proportion of LumB phenotypes, both at the time of the first CTC collection and across all samples (Fig. 3B; Supplementary Fig. S9B). Across all samples, the frequency of LumA and LP phenotypes was similar in mCSPC and mCRPC (Supplementary Fig. S9B). All NE CTC phenotype samples were from patients with histologic NEPC (Fig. 3B; Supplementary Fig. S9B). The rates of prior ARPI therapy were highest in samples with the LumB (72%) and LP (83%) phenotypes (Table 1).

### The Luminal-B CTC Phenotype Is Associated with Adverse Prognosis

We then evaluated the association between CTC phenotype at the time of first CTC collection and overall survival (OS; Fig. 3C). As has been shown previously (45, 46), patients with low CTC burden had the most favorable OS (median OS: not reached). Interestingly, despite higher CTC burden as described above, patients with the LP and LumA CTC phenotypes did not have significantly different outcomes compared with patients with low CTC burden [LP: median OS: 11.8 months, HR: 2.2 (95% CI: 0.6–8.1) and LumA: median OS: 13 months, HR: 2.0 (95% CI: 0.7–6.2)]. By contrast, patients with the LumB and NE CTC phenotype had markedly shorter survival [LumB: median OS: 6 months, HR: 9.1 (95% CI: 3.8–21.8), log-rank *P* < 0.0001 and NE: median OS: 3.7 months, HR: 11.8 (95% CI: 2.4–57.2); log-rank *P* = 0.0019]. In multivariate analysis, mCRPC and NEPC disease status, higher serum PSA, visceral metastasis, CTC content, and LumB and NE CTC phenotypes were all associated with worse prognosis; however, only histologic NEPC and the LumB CTC phenotype were independent prognostic factors (Fig. 3D). The NE CTC phenotype likely was not independently prognostic because of the strong association seen with histologic NEPC (Fig. 3B). Both CTC sample cDNA concentration (as a surrogate for total cellular content) and tumor fraction were similar in the LumA and LumB samples and lower but similar in the LP and NE samples in the survival subset (first CTC collection for each patient; Supplementary Fig. S10A and S10B), similar to what was seen in the highest purity sample from each patient (Supplementary Fig. S5A) and across all samples (Supplementary Fig. S5B). This suggests that the differences in survival seen between high-purity CTC phenotypes are unlikely to be driven by differences in CTC burden.

The identification of a CTC luminal-B–like phenotype associated with equally poor outcomes to the NE CTC phenotype is indicative of a subset of metastatic prostate adenocarcinomas with aggressive disease in the setting of persistent AR signaling. This is in contradistinction to the luminal-A–like phenotype with high AR signaling but low proliferation that is associated with a more favorable prognosis, as well as the novel low AR/low neuroendocrine/low proliferation CTC phenotype that is also associated with more favorable prognosis, unlike the double-negative phenotype that has been described in mCRPC tissue biopsies associated with shorter survival (9). Notably, both the LumB and NE CTC phenotypes were enriched in samples from patients with metastases to the liver, in contrast to the lymph node, bone, and other visceral sites (*P* < 0.005), whereas the LumA CTC phenotype was more common in patients with metastases to the bone rather than visceral disease, and the LP CTC phenotype was seen across metastatic site categories (Fig. 3E). Indeed, the identification of these phenotypes unconfounded by the background of different normal tissues present in tissue biopsies from each metastatic site is a particular strength of the CTC RNA-seq approach.

**Figure 4.**
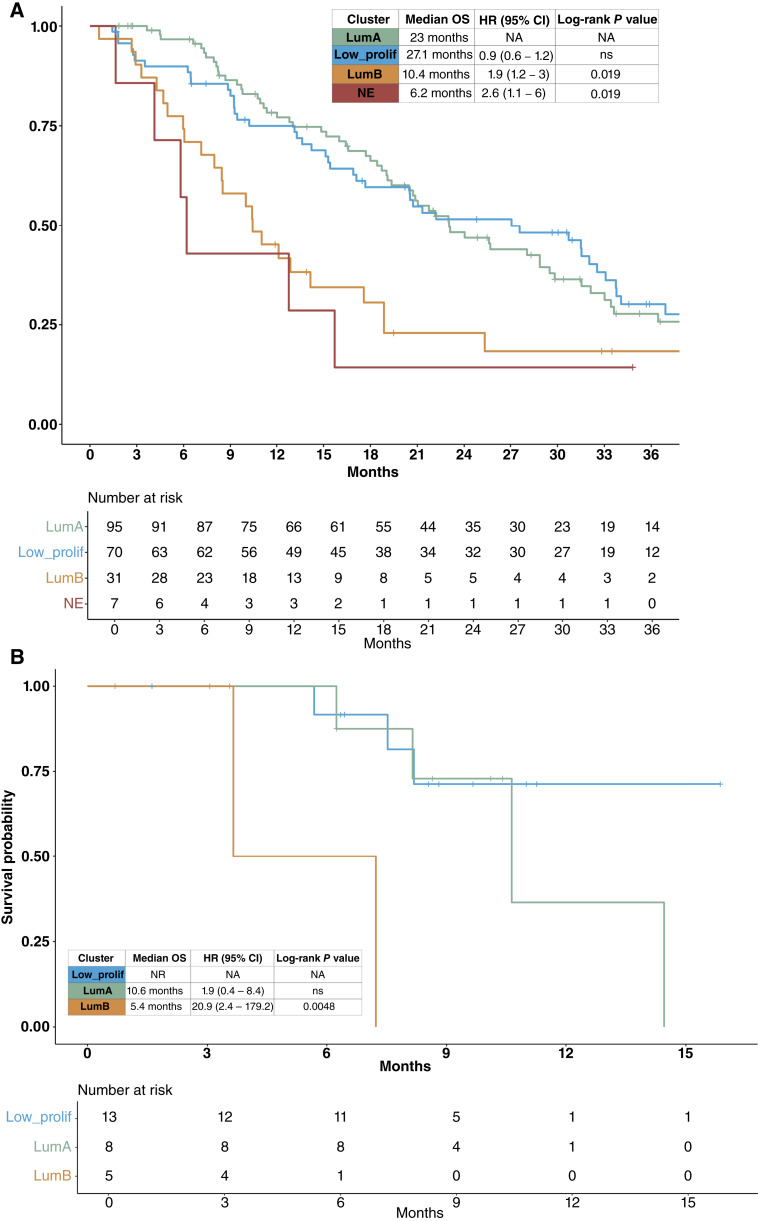
Classification of mCRPC tissue biopsy samples and low-purity CTC samples into the high-purity CTC phenotypes recapitulates survival differences. **A,** Classification of mCRPC tissue biopsy samples (*n* = 203) with a *k*-nearest neighbor (KNN) classifier trained on the high-purity CTC sample phenotypes recapitulates the differences in survival outcome seen between the four CTC phenotypes. **B,** Low CTC burden (Low_CTC) samples with at least minimal epithelial gene expression defined as an expression sum of four epithelial markers (EpCAM, KRT8, KRT18, and KRT19) of >10 transcripts per million (*n* = 30) were reclassified using the same KNN classifier as in **A**. Survival differences between the reclassified Low_CTC samples recapitulates the differences in survival outcome seen between the four CTC phenotypes in the high-purity CTC samples.

To validate the prognostic importance of the transcriptional phenotypes that we identified in the high-purity CTC samples, we used a publicly available mCRPC tissue biopsy RNA-seq dataset that included 203 samples with survival outcomes (11, 47). We used a *k*-nearest neighbors classifier trained on the high-purity CTC sample pathway activity scores to assign the tissue samples to the four CTC phenotypes (low proliferation, LumA, LumB, or neuroendocrine) and found that survival outcomes for these phenotypes in the tissue dataset recapitulated the differences seen in the CTC dataset (Fig. 4A). In addition to externally validating the prognostic value of the CTC phenotypes, this result also provides evidence that the transcriptional phenotypes in our high-purity CTC samples mirror transcriptional patterns found in tissue biopsies.

We then evaluated whether the same *k-*nearest neighbors classifier would be able to reclassify low-purity CTC samples that had evidence of epithelial/CTC content but did not have a sufficiently high tumor fraction to be included in the high-purity analysis (*n* = 30). Indeed, applying the classifier to single-sample pathway scores from these samples resulted in the reclassification of 13 samples as low proliferation, six samples as LumA, and five samples as LumB. The OS in the low-purity LumB classified samples [median OS: 5.4 months, HR: 20.9 (95% CI: 2.4–179.2), log-rank *P* = 0.0048] was significantly shorter than those classified as LumA (median OS: 10.6 months) or LP (median OS: not reached; Fig. 4B), similar to patients in the high-purity LumB group.

Finally, using canonical AR signaling and neuroendocrine gene signatures (7) that were held out from the initial pathway clustering so that they could be used for independent assessment, we evaluated differences in AR signaling and neuroendocrine differentiation between the CTC phenotypes. We confirmed that AR signaling was highest in LumA and LumB samples, intermediate in LP samples, and very low in NE samples (Supplementary Fig. S11A). By contrast, neuroendocrine scores were much higher in the NE samples than in the LumA, LumB, and LP samples; however, the LumB samples also had modestly but significantly higher neuroendocrine scores than the LumA and LP samples (Supplementary Fig. S11B). Pathways associated with *RB1* loss and *PTEN* loss signatures were highest in NE samples, although they were also elevated in the LumB samples (Supplementary Fig. S11C and S11D). However, in contrast to the NE samples, only very few of the LumB samples expressed transcription factors associated with terminal NEPC differentiation including *INSM1* and *ASCL1* (Supplementary Fig. S7A–S7D).

### The Luminal-B CTC Phenotype Is Associated with Early Progression on ^177^Lu–PSMA-617

**Figure 5.**
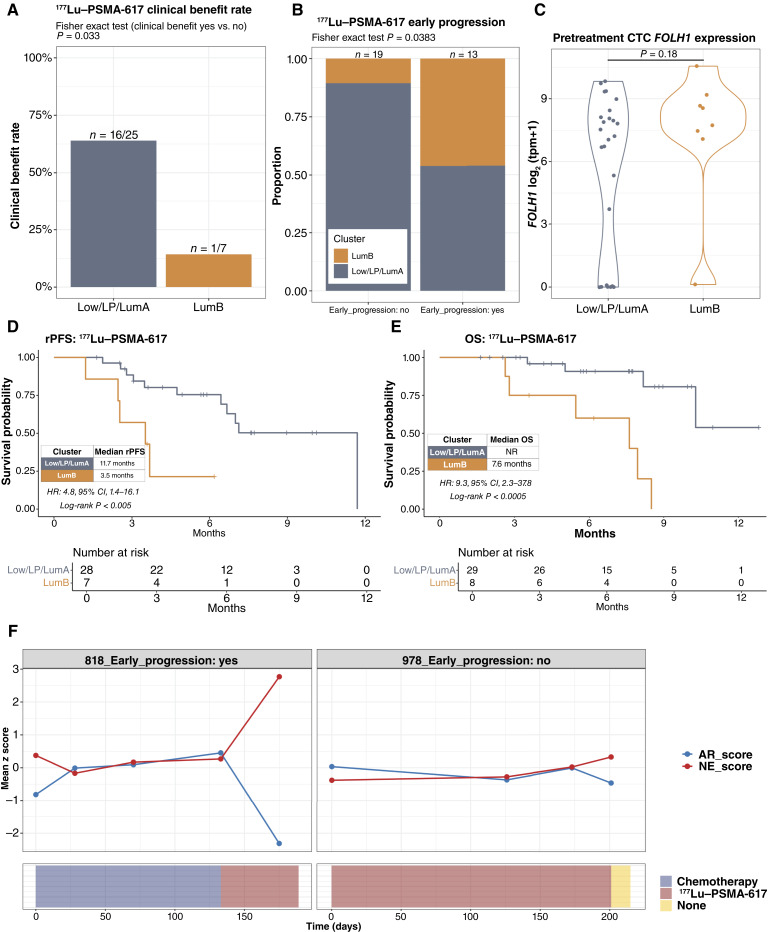
Luminal-B CTC phenotype and persistent PSMA expression are associated with poor response to ^177^Lu–PSMA-617. **A,** Clinical benefit rate (the best radiographic response of stable disease, partial response, or complete response) for ^177^Lu–PSMA-617 in patients with favorable pretreatment CTC phenotypes (Low_CTC, LP, and LumA) vs. pretreatment LumB phenotype. **B,** Proportion of pretreatment LumB vs. favorable CTC phenotypes for patients with and without ^177^Lu–PSMA-617 early progression (disease progression within the first three cycles/18 weeks of treatment). **C,** Baseline CTC *FOLH1* (PSMA) gene expression for LumB (*n* = 8) vs. favorable CTC phenotypes (*n* = 29). Kaplan–Meier plot for (**D**) radiographic progression-free survival (rPFS) and (**E**) OS illustrates decreased rPFS and OS after starting ^177^Lu–PSMA-617 for patients with the pretreatment LumB CTC phenotype. **F,** Longitudinal evaluation of consensus AR/luminal and neuroendocrine pathway scores during ^177^Lu–PSMA-617 treatment. Patient 818 (left) represents an example of a patient with early progression on ^177^Lu–PSMA-617 who demonstrated a rapid increase in NE score and decrease in AR score on treatment. Patient 978 (right) illustrates a patient with a longer duration of response who also shows an increased NE score and decreased AR score at the time of disease progression.

In the CTC cohort, 37 patients receiving standard-of-care ^177^Lu–PSMA-617 (Supplementary Table S1) were enrolled in a prospective substudy with longitudinal CTC collections at pretreatment, cycle 2, cycle 4, cycle 6, and disease progression. In the pretreatment samples, eight of 37 patients had a LumB CTC phenotype, and none had an NE CTC phenotype (Supplementary Fig. S12A). In the 32 patients evaluable for this endpoint, we found that the clinical benefit rate was significantly higher in patients with favorable pretreatment CTC phenotypes (Low_CTC, LP, or LumA) than in patients with pretreatment LumB CTC phenotype (64% vs. 14%; *P* = 0.033; Fig. 5A). Conversely, the LumB CTC phenotype was enriched in pretreatment samples from patients who experienced disease progression within the first three cycles/18 weeks of ^177^Lu–PSMA-617 treatment (early progression) compared with those who did not (Fig. 5B). Pretreatment serum PSA levels (Supplementary Fig. S12B) were similar between LumB versus non-LumB groups, as were CTC *FOLH1* (PSMA) gene expression (Fig. 5C; Supplementary Fig. S12C) and PSMA PET-CT avidity (Supplementary Fig. S12D), suggesting that the higher rates of early progression and lower likelihood of clinical benefit seen in patients with the LumB CTC phenotype were not driven by a lack of target expression. We also evaluated the relationship between PSMA PET-CT avidity, CTC *FOLH1* expression, and CTC phenotype for patients with available contemporary PSMA PET-CT imaging in our overall cohort. We found concordant high CTC *FOLH1* expression in CTC samples from patients with PSMA PET-CT avidity (highest SUVmax) in the highest quartile (Supplementary Fig. S13A). There was no significant difference in PSMA PET-CT highest SUVmax between CTC phenotypes (Supplementary Fig. S13B), although this analysis was limited by the small number of samples with matched PSMA PET-CT imaging. Finally, with a median follow-up of 7.6 months, we demonstrated that patients with the pretreatment LumB CTC phenotype had both decreased radiographic progression-free survival [3.5 vs. 11.7 months, HR: 4.8 (95% CI: 1.4–16.1), log-rank *P* < 0.005; Fig. 5D] and OS [7.6 months vs. NR, HR: 9.3 (95% CI: 2.3–37.8), log-rank *P* < 0.0005; Fig. 5E] compared with patients with the more favorable pretreatment CTC phenotypes.

Taken together, these data suggest that ^177^Lu–PSMA-617 therapy is not able to overcome the poor prognosis associated with the LumB CTC phenotype despite similar levels of *FOLH1*/PSMA expression as the more favorable CTC phenotypes. We therefore investigated other factors that could contribute to the lack of ^177^Lu–PSMA-617 response in the LumB CTC phenotype group. *MYC* activity has previously been associated with a luminal but highly proliferative luminal-B–like phenotype in tissue biopsies (41), and we found that Hallmark *MYC* signaling signatures were significantly higher in the LumB versus LumA samples in both the high-purity CTC cohort and our validation tissue biopsy cohort (Supplementary Fig. S14A–S14D). *MYC* overexpression has previously been associated with radioresistance in prostate cancer (48) and other solid tumors (49, 50). To further address the possibility that the LumB CTC phenotype could be associated with payload/radioresistance, we evaluated the PORTOS prostate cancer radiation response signature (51). Intriguingly, we observed that PORTOS scores were lower in LumB vs. LumA samples in both the CTC and tissue cohorts (Supplementary Fig. S14E and S14F), which would be consistent with less benefit for a radioligand therapy in the LumB phenotype. Although these findings are highly exploratory, they are suggestive of a role of payload response and warrant further investigation in additional prospective cohorts.

### Longitudinal CTC Transcriptional Profiling Demonstrates Evolving Neuroendocrine versus AR Signaling Activity during ^177^Lu–PSMA-617 Radioligand therapy

We leveraged the unique capability of liquid biopsies for serial longitudinal sampling to interrogate the evolution of CTC *FOLH1* expression, AR signaling, and neuroendocrine signature scores in patients on treatment with ^177^Lu*–PSMA-617*. CTC *FOLH1* expression remained high in post-progression samples from patients who had experienced early progression within the first three cycles of treatment (Supplementary Fig. S15A). By contrast, we observed moderately lower *FOLH1* expression post-progression than in pretreatment samples from patients who responded to ^177^Lu–PSMA-617 (Supplementary Fig. S15A). We generated consensus AR/luminal and neuroendocrine pathway scores for each sample from a set of established AR/luminal pathway scores and neuroendocrine pathway scores (Supplementary Table S2). The slope of change in AR and NE scores over time was then quantified for the 18 patients in the ^177^Lu–PSMA-617 cohort with multiple high-purity CTC samples suitable for pathway analysis. Although some patients had stable AR/NE scores over time on treatment (Supplementary Fig. S15B), five patients in this group demonstrated a decrease in AR score and increase in NE score over time on ^177^Lu–PSMA-617 of whom four of five were patients with early progression within the first three cycles as defined above (Fig. 5F; Supplementary Fig. S15C). Patient 818 was a patient with early progression who had transitioned from prior chemotherapy to ^177^Lu–PSMA-617 with a subsequent rapid and marked increase in NE score and decrease in AR score (Fig. 5F), whereas patient 978, the single patient without early progression in this group, experienced an increased NE score and decrease in AR score at the time of disease progression on ^177^Lu–PSMA-617 (Fig. 5F). These observations suggest that CTC transcriptional profiling could be used to understand how AR and NE components are dynamically changing under the selective pressure of ^177^Lu–PSMA-617 with androgen deprivation therapy.

### Cell-Surface Target Expression across CTC Phenotype Clusters

**Figure 6.**
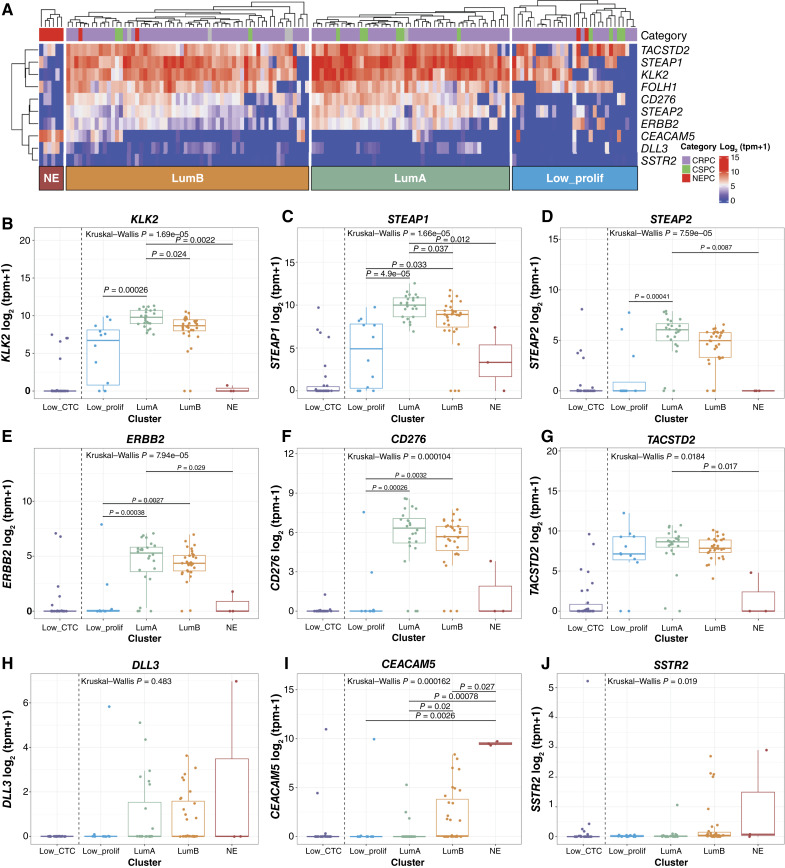
Cell-surface target expression is variable across CTC phenotypes. **A,** Heatmap of gene expression of cell-surface targets ordered by clustering in Fig. 1D. **B–J,** Cell-surface target expression by CTC phenotype for targets associated with prostate adenocarcinoma (*KLK2*, *STEAP1*, and *STEAP2*), pan-cancer cell-surface targets expressed in prostate cancer including *TACSTD2* (TROP2), *ERBB2* (HER2), and tumor immune checkpoint cell-surface protein *CD276* (B7H3), as well as targets associated with prostate neuroendocrine differentiation (*DLL3*, *CEACAM5*, and *SSTR2*). For patients with multiple CTC samples, the highest purity sample is included (Low_CTC, *n* = 30; Low_prolif, *n* = 12; LumA, *n* = 24; LumB, *n* = 31; and NE, *n* = 3). Low_CTC samples are included for reference but not included in statistical comparisons because they are expected to have low/no expression due to low CTC content.

Given the clear need for novel therapeutic approaches for the LumB CTC phenotype, we sought to leverage CTC RNA-seq to understand the expression of cell-surface targets beyond PSMA across the CTC phenotype clusters. A survey of cell-surface targets with agents in development in prostate cancer revealed variable expression patterns in CTCs (Fig. 6A). Interestingly, canonical prostate adenocarcinoma cell-surface targets *KLK2* and *STEAP1* had significantly lower expression in the LumB versus LumA samples (Fig. 6B and C; Supplementary Fig. S16A and S16B), and *STEAP2* (Fig. 6D; Supplementary Fig. S16C) also had a trend toward lower expression in LumB, in contrast to *FOLH1*, which was highly expressed in both luminal CTC phenotypes (Fig. 2E; Supplementary Fig. S6D). The expression of *STEAP2* as well as pan-cancer targets *ERBB2* (HER2) and the tumor immune checkpoint cell-surface protein *CD276* (B7H3; Fig. 6D–F; Supplementary Fig. S16C–S16E) seemed restricted to the LumA and LumB samples, in contrast to *KLK2*, *STEAP1*, *FOLH1*, and epithelial cell-surface protein *TACSTD2* (TROP2; Fig. 6G; Supplementary Fig. S16F), which were also expressed in the LP samples. Finally, the expression of *DLL3*, *CEACAM5*, and *SSTR2*, which are targets associated with neuroendocrine differentiation, was significantly higher in the NE CTC phenotype samples and was also expressed in lower levels in a subset of LumA and LumB samples (Fig. 6H–J; Supplementary Fig. S16G–S16I).

We have previously shown concordance between transcriptional and protein expression of cell-surface target protein in tissue biopsies (52), but given the differences in transcriptional expression between CTC phenotypes for some of the cell-surface targets, as well as the observation of transcriptional expression of targets associated with neuroendocrine differentiation in a subset of non-NE CTC samples, we performed single CTC immunofluorescent protein expression quantification of DLL3 or PSMA protein in matched samples available for a subset of patients.

Although we did not identify any samples with uniformly DLL3-positive CTCs, the samples with the highest number of DLL3-positive CTCs in single CTC protein quantification also had DLL3 detected at the transcriptional level and both of these samples had a luminal-B CTC phenotype (Supplementary Fig. S17A and S17B). The remainder of the CTC samples in this cohort had low or no DLL3 expression at either the protein or transcriptional level, including examples of both adenocarcinoma and histologic NEPC (Supplementary Fig. S17B). Importantly, the single-cell protein quantification identified intrasample heterogeneity, with a small number of DLL3-positive CTCs even in samples with primarily DLL3-negative/low CTCs (Supplementary Fig. S17B).

We identified samples with primarily PSMA-positive/high CTCs in single CTC protein quantification that also had high expression at the transcriptional level, and conversely, samples with primarily PSMA-negative/low protein expression that also had low transcriptional expression (Supplementary Fig. S17C and S17D). Both luminal-A and luminal-B phenotype samples were represented among samples with primarily PSMA-positive/high CTCs. However, as with DLL3, we observed significant heterogeneity in PSMA protein expression at the single-cell level, and in some samples, we saw both PSMA-positive/high and PSMA-negative/low CTCs, associated with more variable transcriptional expression (Supplementary Fig. S17D).

## Discussion

We report in this study the largest CTC RNA-seq cohort of patients with metastatic prostate cancer and demonstrate that CTC RNA-seq identifies prostate cancer lineage states associated with prognosis, validated in an independent external tissue biopsy cohort. This includes a CTC NE phenotype concordant with tissue histology and associated with markedly inferior OS. Importantly, we identified a more common LumB CTC phenotype transcriptionally defined by persistent AR signaling activity and high proliferation and associated with an equally poor prognosis as NEPC. The LumB CTC phenotype was enriched after ARPI exposure and associated with visceral metastasis, a known poor prognosis clinical feature (53). Gene signatures associated with proliferation and *RB1* and *PTEN* loss are high in the LumB phenotype, but in contrast to the NE phenotype, it does not express NEPC-associated transcription factors and does maintain evidence of AR activity, both transcriptionally and with much higher serum PSA levels than the NE phenotype samples. Indeed, serum PSA levels are similar between the LumA and LumB phenotype samples, despite a striking difference in prognosis. This highlights the power of CTC transcriptional phenotyping to identify an aggressive subset of non-neuroendocrine mCRPC that remains persistently androgen driven but nevertheless is associated with poor clinical outcomes. We also find that this persistently androgen-driven but poor prognosis phenotype is also associated with lower clinical benefit rate and early progression with ^177^Lu–PSMA-617, independent of *FOLH1*/PSMA expression, which is similar between the luminal phenotypes.

The ability to interrogate distinct molecular features is critical for prostate cancer biomarker development, given the divergent mechanisms of resistance that extend beyond genomic alterations. Although cfDNA profiling can provide DNA variant identification (54, 55) and indirect inference of epigenetic regulation of gene expression (21, 56, 57), the direct and comprehensive transcriptional profiling of CTCs has the potential to provide a platform that can match the capabilities of tissue profiling. Transcriptomic phenotyping can now be performed at scale using this platform without barriers to obtaining tissue samples such as safety and accessibility to biopsy site, as well as the technical challenges in obtaining high-quality tissue from bone biopsies. Although CTC sampling does not allow for simultaneous assessment of the tissue microenvironment, high-purity CTC samples, which we were able to obtain in the majority of the patients in our cohort, allow for isolation of tumor cell populations shed from multiple metastatic sites without the variability of the nontumor component of the sample that would be seen with tissue biopsies from different sites. Additionally, the ability to perform longitudinal monitoring of CTC transcriptional phenotypes provides opportunities to noninvasively monitor for and detect lineage state transitions to guide therapeutic interventions. Finally, we show that transcriptional profiling of CTCs can be leveraged to identify target expression across lineage/transcriptional states for cell-surface targeted agents currently in clinical development, providing the opportunity to evaluate for potential new therapeutic strategies to target treatment-resistant disease.

A key limitation of our study is that CTCs may not be perfect surrogates for metastatic tissue and may not fully capture the heterogeneity between different metastatic sites. However, we were able to validate the prognostic associations of our CTC phenotypes in an independent tissue biopsy RNA-seq cohort which included multiple metastatic biopsy sites, suggesting that they do reflect tumor tissue phenotypes. Bulk rather than single-cell RNA-seq of CTCs allows higher throughput as well as reduced dropout of low-abundance transcripts but does not allow deconvolution of single-cell heterogeneity in tumor phenotypes; future studies can incorporate both parallel single CTC protein phenotyping and single CTC RNA-seq in select high-CTC-burden samples to address this question. Finally, a clinical limitation of our study is that although sample collection was prospective, patients were not assigned to specific treatments, and there was no randomization. This cohort therefore represents real-world treatment patterns but is subject to possible selection bias and unmeasured confounding factors beyond those included in our multivariate analysis. However, based on our initial results, a prospective validation study cohort is ongoing for patients starting ARPI therapy for mCRPC (NCT06141993) who will undergo CTC RNA-seq at baseline, on treatment, and at disease progression. Additional clinical use studies are ongoing or preparing to begin enrollment to evaluate evolution and prognostic and predictive value of the CTC transcriptional phenotypes in patients receiving metastasis directed therapy for oligometastatic CSPC (NCT05156905), novel targeted therapy combinations for mCRPC (NCT05502315, NCT06150417, and NCT06632977), and somatostatin receptor–targeted radioligand therapy for NEPC (NCT05691465). Incorporation of these methods longitudinally into prospective clinical trials in metastatic prostate cancer will speed the development of both predictive biomarkers as well as on-treatment biomarkers for earlier response evaluation and molecular mechanisms of treatment resistance. Future directions outside of prostate cancer include testing of these methods across multiple additional malignancies and therapeutic modalities, with trials ongoing or in development including those in metastatic breast cancer (NCT04762979 and NCT06099769) and renal cell carcinoma (NCT05327686), among others.

## Methods

### Multi-institutional Cohort

Patients were enrolled under an institutional review board–approved biospecimen protocol (1202-1214) for CTC analysis. Written informed consent was obtained from all participants prior to enrollment. The study was conducted in compliance with the Declaration of Helsinki. Blood samples were collected prospectively from 117 patients with metastatic prostate cancer who were treated at the University of Wisconsin Carbone Cancer Center, William S. Middleton Memorial Veterans Hospital, University of California San Diego Moores Cancer Center, or Dana-Farber Cancer Institute between September 2022 and June 2024. Study data were managed using approved REDCap electronic data tools (RRID: SCR_003445) hosted at the University of Wisconsin–Madison, School of Medicine and Public Health (58).

### CTC Isolation and RNA Extraction from Patient Blood Samples

Whole blood (15–30 mL) was collected from patients with metastatic prostate cancer in BD vacutainer EDTA tubes (Becton Dickinson, cat. #366643). Samples from other institutions were shipped in temperature-controlled packaging, and all samples were processed within 36 hours of collection. Mononuclear cells were isolated with a Ficoll-Paque PLUS (Cytiva, cat. #17144003) gradient before undergoing depletion with CD45 and CD235a MACS beads by following the manufacturer’s suggested methods (Miltenyi Biotec, cat. #130-045-801 and 130-050-501). The multiplexed Technology for Automated Extraction(mTAE) platform, as described previously, was used for the live cell capture of CTCs using an anti-EpCAM-biotin antibody (R&D Systems, cat. #BAF960, RRID: AB_356818) conjugated to SeraMag SpeedBeads Streptavidin-Blocked magnetic particles (Cytiva, cat. # 21152104010150; ref. 23). The extraction of mRNA is integrated on the automated mTAE platform as previously described, integrating Oligo(dT) magnetic particles from the Dynabeads mRNA DIRECT Purification Kit (Thermo Fisher Scientific, cat. #61012; refs. 28, 59).

### Library Preparation

Extracted mRNA was converted to double-stranded cDNA using the SMART-seq mRNA kit (Takara Bio, cat. # 634773) using 10.5 µL of isolated RNA. After cDNA amplification, the product was purified using 1:1 volume of NucleoMag NGS cleanup beads (Takara Bio, cat. # 744970.50). We assessed cDNA quality and yield using the High Sensitivity D5000 ScreenTape Assay (Agilent, cat #5067-5588) on the 4200 TapeStation System (Agilent, RRID: SCR_018435). Libraries were prepared using a miniaturized version of the Nextera XT DNA library preparation kit (Illumina, cat. #FC-131-1096) using 25% of the suggested kit volumes. About 1.25 µL of 100 pg/µL cDNA (or less if cDNA yield <100 pg/ µL) was used for each library preparation. Library cleanup was performed with individual or pooled PCR with 0.8:1 NucleoMag NGS cleanup beads, and libraries were quantified on a Thermo Fisher Scientific Qubit 4.0 Fluorometer (FC-131-1096) using the 1× dsDNA High Sensitivity Assay Kit (Thermo Fisher Scientific, cat. #Q33231). Size distribution was assessed using either High Sensitivity D1000 ScreenTape System (Agilent, cat. #76645-162) or D1000 ScreenTape (Agilent, cat. #76645-180) on the 4200 TapeStation System (Agilent). We used the University of Wisconsin–Madison Biotechnology Center’s DNA Sequencing Facility (Research Resource Identifier—RRID: SCR_017759) for paired-end sequencing (2 × 150) with a target depth of 50 million reads. Reads were aligned using the STAR (RRID: SCR_004463) aligner (60), and gene abundance was calculated using featureCounts (RRID: SCR_012919; ref. 61).

### Inferring CTC Sample Tumor Purity

To evaluate CTC sample tumor purity using the ESTIMATE algorithm (29), immune, stromal, and combined ESTIMATE scores were calculated as published. We found that inferred tumor purity from CTC sample ESTIMATE scores was higher than expected using the original ESTIMATE conversion equation (Supplementary Fig. S1A), likely because of differences in immune composition of the CTC versus tissue samples (Supplementary Fig. S1B). After re-solving the conversion equation using control LNCaP prostate tumor cell line versus peripheral blood mononuclear cell samples, the adjusted inferred tumor content mirrored the expected distribution (Supplementary Fig. S1C). LNCaP cells (RRID: CVCL_1379) were a gift from David Jarrard (University of Wisconsin- Madsion) and were sourced from ATCC without reauthentication after purchase. They were *Mycoplasma* tested in the same month as that of the assessment using the EZ-PCR Mycoplasma Detection Kit (Sartorius, cat. #20-700-20) and were used within 15 passages of thawing. The CIBERSORTx tool (RRID: SCR_016955) with a published peripheral blood mononuclear cell signature matrix (30) was used as an orthogonal approach to confirm immune content, and therefore tumor content as immune cells were the predominant benign tissue background in CTC samples.

### Pathway Analysis and Phenotype Clustering

To robustly select samples with high enough tumor purity for pathway analysis, we elected for a conservative threshold of >40% inferred tumor purity. Additionally, as a secondary filter for epithelial/tumor content, we required samples to have an expression sum of four epithelial markers (EpCAM, KRT8, KRT18, and KRT19) of >1,500 transcripts per million, and as a secondary filter for immune content, we required a CIBERSORTx absolute immune score of <75. Molecular Signatures Database hallmark pathway gene sets were downloaded from the Molecular Signatures Database (RRID: SCR_016863). Prostate cancer gene sets were manually curated from the published literature (Supplementary Material S1). Pathway activity was calculated using AUCell (RRID: SCR_021327; ref. 62) with a threshold of 10%. Consensus K-means clustering of samples was performed on scaled single-sample pathway activity scores to identify distinct transcriptional phenotype clusters. In order to calculate PORTOS scores, we first normalized the CTC and metastatic tissue gene expression data in our study to the original training dataset of PORTOS (GSE46691; ref. 63) using COMBAT (64). PORTOS scores were then calculated using the same model as in the original publication (51) on the batch-corrected gene expression values in the CTCs and metastatic tissue datasets. For longitudinal analysis of AR and NE signaling during ^177^Lu–PSMA-617 treatment, we generated a single-consensus AR and NE score for each sample by averaging a set of established AR/luminal pathway scores and neuroendocrine pathway scores (Supplementary Table S2) to minimize differences due to any individual gene set. For each subject, the change in AR/NE score was quantified as the slope of the linear regression of AR or NE consensus scores across ^177^Lu–PSMA-617 longitudinal CTC collection timepoints. All pathway and clustering analyses were completed in R version 4.2.2 [The R Project for Statistical Computing (RRID: SCR_001905)].

### Phenotype Cluster Classification in Low-Burden CTC and Metastatic Tissue Samples

Single-sample pathway activity scores were calculated as described above for 203 publicly available mCRPC tissue biopsy RNA-seq samples with available OS outcomes (11) and for low CTC burden (Low-CTC) samples in our cohort with at least minimal epithelial gene expression defined as an expression sum of four epithelial markers (EpCAM, KRT8, KRT18, and KRT19) of >10 transcripts per million. Classification of these samples into the high-purity CTC transcriptional phenotypes using scaled single-sample pathway scores was performed with the class package (65) in R version 4.2.2, using a *k*-nearest neighbor model trained on the scaled pathway scores of the high-purity CTC samples.

### PSMA PET-CT Analysis

Where PSMA PET-CT imaging study results were available within 18 weeks of a CTC collection with at least minimal epithelial gene expression as defined above, the SUVmax for all measured lesions, physiologic liver SUVmean, and SUVmax as reported by the reading radiologist were extracted from the imaging report. For evaluation of PSMA PET-CT lesion avidity across CTC phenotype clusters and correlation between PSMA PET-CT avidity and CTC *FOLH1* expression, as well as pretreatment PSMA PET-CT avidity in the ^177^Lu–PSMA-617 substudy, the highest lesion SUVmax for each imaging study was selected and normalized to physiologic liver SUVmax to facilitate comparison across images.

### PSMA and DLL3 Single CTC Protein Phenotyping

Details of VERSA manufacturing and operation are described by Sperger and colleagues (66). Whole blood (7.5 mL) was collected in BD vacutainer EDTA tubes (Becton Dickinson, cat. # 366643) or in CellSave tubes (Menarini Silicon Biosystems). Samples from other institutions were shipped in temperature-controlled packaging, and all samples were processed within 36 hours of collection. Mononuclear cells were isolated as above using the Ficoll-Paque PLUS gradient (GE Healthcare) followed by fixation using BD Cytofix (BD Biosciences). CTCs were isolated by using VERSA, using an antibody against EpCAM (R&D Systems, cat. #BAF960, RRID: AB_356818) conjugated to Sera-Mag SpeedBeads Streptavidin-Blocked magnetic particles (Cytiva, cat. #21152104010150). CTC samples protein expression was assessed by immunofluorescence staining. The following antibodies were used to identify CTCs: Exclusion [anti-CD45 (BioLegend, cat. #304018, RRID: AB_389336), anti-CD34 (BioLegend, cat. #343508, RRID: AB_1877133), anti-CD66b (BioLegend, cat. #305109, RRID: AB_2563170), anti-CD14 (BioLegend, cat. #325612, RRID: AB_830685), anti-CD27 (BioLegend, cat. #302812, RRID: AB_493082), anti-CD16 (BioLegend, cat. #360714, RRID: AB_2563021)], anti-pan-cytokeratin (Santa Cruz Biotechnology, cat # sc-8018 AF790, RRID: AB_627396), and Hoechst 33342 (Thermo Fisher Scientific). To assess extracellular proteins, we used PE anti-DLL3 antibody (EPR22592-18; Abcam, cat. # ab305808) or PE anti-human PSMA (FOLH1) antibody (BioLegend, cat. # 342504, RRID: AB_2247193). Extracellular antibodies were stained at 4°C for 30 minutes. For intracellular staining, cells were permeabilized, stained, and washed with BD Perm/Wash Buffer. Images were taken with a 10× objective using the Nikon Eclipse Ti-E with an ORCA-Flash4.0 V2 Digital CMOS camera (Hamamatsu Photonics) and NIS-Elements Advanced Research microscope imaging software (Nikon Instruments, RRID: SCR_014329). Images were background subtracted, and Hoechst-positive cell identification followed by single-cell mean fluorescence intensity measurements were performed using the NIS-Elements Advanced Research microscope imaging software. Single-cell mean fluorescent intensities for each channel were 50% quantile normalized (67), followed by CTC identification as pan-cytokeratin positive, exclusion (CD45/CD34/CD66b/CD14/CD27/CD16) negative cells. Single-cell PSMA and DLL3 protein expression was quantified as log10-transformed mean fluorescence intensity (logMFI), and CTCs were considered to be positive for the protein if logMFI was above the 95th percentile of logMFI in non-CTCs across all samples for each assay.

### Statistical Analysis

For comparisons between clusters, a Fisher exact test was used for categorical variables and either a Mann–Whitney *U* test or Kruskal–Wallis test with a Dunn test for multiple comparisons was used for continuous variables, as appropriate. For patients with multiple CTC samples available, the highest purity sample from each patient was used; however, for clinical variables related to survival outcomes between clusters, the first CTC collection for each patient was used to align with the survival analysis. The OS was defined as the date of death or that censored at last contact relative to the first CTC sample collection or at ^177^Lu–PSMA-617 treatment start as appropriate. The clinical benefit rate was defined as the percentage of patients with best radiographic response of stable disease, partial response, or complete response according to RECIST 1.1 criteria during ^177^Lu–PSMA-617 treatment. Early progression during ^177^Lu–PSMA-617 treatment was defined as discontinuation of therapy within 18 weeks (three cycles) after the first dose due to radiographic and/or PSA progression as assessed by the treating physician. Radiographic progression-free survival was defined as investigator-assessed radiographic progression according to RECIST 1.1 criteria for soft tissue and lymph node disease and PCWG3 for bone disease or death due to any cause or censored at the date of last disease assessment relative to ^177^Lu–PSMA-617 treatment start. The Kaplan–Meier method was used to estimate the survival distributions by clusters, and log-rank test was used to compare groups. Multivariate Cox proportional hazards models were fitted to quantify the association of molecular and clinical variables with the OS. All statistical analyses were completed in R version 4.2.2 (R Foundation for Statistical Computing).

### Data Availability

Our institutional protocol does not allow unrestricted public access to the raw sequencing data. Therefore, data sharing requests must be submitted to the University of Wisconsin–Madison for review and approval.

## Supplementary Material

Table S1 Table S1. Clinical characteristics of the 177LuPSMA-treated cohort

Table S2 Table S2. Signature scores for longitudinal AR/luminal versus NE analysis

Figure S1 Figure S1. Calibration of ESTIMATE tumor content prediction.

Figure S2 Figure S2. RNA sequencing quality metrics

Figure S3 Figure S3. Reproducibility of tumor/immune content assessment, gene expression and pathway scores.

Figure S4 Figure S4. Sample processing is not different between CTC phenotypes.

Figure S5 Figure S5. Tumor fraction and immune content across CTC phenotypes.

Figure S6 Figure S6. Expression of epithelial and prostate adenocarcinoma genes across CTC phenotypes for all samples.

Figure S7 Figure S7. Expression of neuroendocrine genes across CTC phenotypes.

Figure S8 Figure S8. MKI67 expression in each CTC phenotype cluster for all samples.

Figure S9 Figure S9. Serum PSA and disease category by CTC phenotype for all samples.

Figure S10 Figure S10. Sample cDNA concentration and tumor fraction by CTC phenotype in the survival analysis subset.

Figure S11 Figure S11. Luminal B phenotype is associated with activation of pathways associated with RB1 and PTEN loss.

Figure S12 Figure S12. Pretreatment CTC and PSMA-PET characteristics in the 177Lu-PSMA-617 sub-study cohort.

Figure S13 Figure S13. CTC FOLH1 expression and transcriptional phenotype and PSMA-PET characteristics.

Figure S14 Figure S14. Luminal B phenotype is associated with higher MYC signaling and lower radiation response scores.

Figure S15 Figure S15. Longitudinal FOLH1 expression and pathway analysis of CTCs in the 177Lu-PSMA-617 sub-study cohort.

Figure S16 Figure S16. Cell surface target expression across CTC phenotype clusters for all samples.

Figure S17 Figure S17. Concordance between protein and RNA expression of cell surface targets in prostate CTCs.

Supplementary Genesets Supplementary Genesets includes prostate cancer specific genesets curated from published literature
