# Supplementary material for: High-Purity CTC RNA Sequencing Identifies Prostate Cancer Lineage Phenotypes Prognostic for Clinical Outcomes
Source: Cancer Discov. Author manuscript; Available in PMC 2025 May 3. (PMC12046329; doi:10.1158/2159-8290.CD-24-1509)
Supplement: Table S1 [file NIHMS2074075-supplement-Table_S1.pdf]

Table S1. Clinical characteristics of the 177LuPSMA-treated cohort

Characteristic	177LuPSMA
**No. of patients**	37
**Age, median (range), years**	72 (56-86)
**PSA at blood draw median (range), ng/mL**	58 (0.2-1896)
**Gleason Score, n (%)**	
**≤6**	1 (3)
**7**	3 (8)
**8**	7 (18)
**≥9**	21 (55)
**Unknown**	6 (16)
**De novo metastatic disease, n (%)**	
**Yes**	25 (66)
**No**	13 (34)
**Unknown**	0 (0)
**Metastatic sites, n (%)**	
**Lymph node**	26 (70)
**Bone**	36 (97)
**Non-liver Visceral**	7 (18)
**Liver**	9 (24)
**Unknown**	0(0)
**Type of disease, n (%)**	
**CSPC**	(0)
**CRPC**	36 (97)
**NEPC**	1 (3)
**Prior Treatment, n (%)**	
**ARPI**	36 (97)
**Chemotherapy**	31 (83)

Abbreviations: ARPI, Androgen Receptor Pathway Inhibitor, CSPC, Castration Sensitive Prostate Cancer; CRPC, Castration Resistant Prostate Cancer; NEPC, Neuroendocrine Prostate Cancer
