# Supplementary material for: High-Purity CTC RNA Sequencing Identifies Prostate Cancer Lineage Phenotypes Prognostic for Clinical Outcomes
Source: Cancer Discov. Author manuscript; Available in PMC 2025 May 3. (PMC12046329; doi:10.1158/2159-8290.CD-24-1509)
Supplement: Table S2 [file NIHMS2074075-supplement-Table_S2.pdf]

Table S2. Signature scores for longitudinal AR/luminal versus NE analysis

Group	Signature	Reference
**AR/Luminal**	Hieronymus_AR_signaling	Hieronymus et al., 2006
**AR/Luminal**	AR_FHCRC	Labreque et al., 2019
**AR/Luminal**	Beltran_NEPC_DN	Beltran et al., 2016
**AR/Luminal**	Aggarwal_NEPC_DN	Aggarwal et al., 2018
**AR/Luminal**	Zhang_luminal	Zhang et al., 2016
**NE**	Beltran_NEPC_UP	Beltran et al., 2016
**NE**	NE_FHCRC	Labreque et al., 2019
**NE**	Lundberg_NE	Lundberg et al., 2023
**NE**	Aggarwal_NEPC_UP	Aggarwal et al., 2018
