# Supplementary material for: High-Purity CTC RNA Sequencing Identifies Prostate Cancer Lineage Phenotypes Prognostic for Clinical Outcomes
Source: Cancer Discov. Author manuscript; Available in PMC 2025 May 3. (PMC12046329; doi:10.1158/2159-8290.CD-24-1509)
Supplement: Figure S1 [file NIHMS2074075-supplement-Figure_S1.pdf]

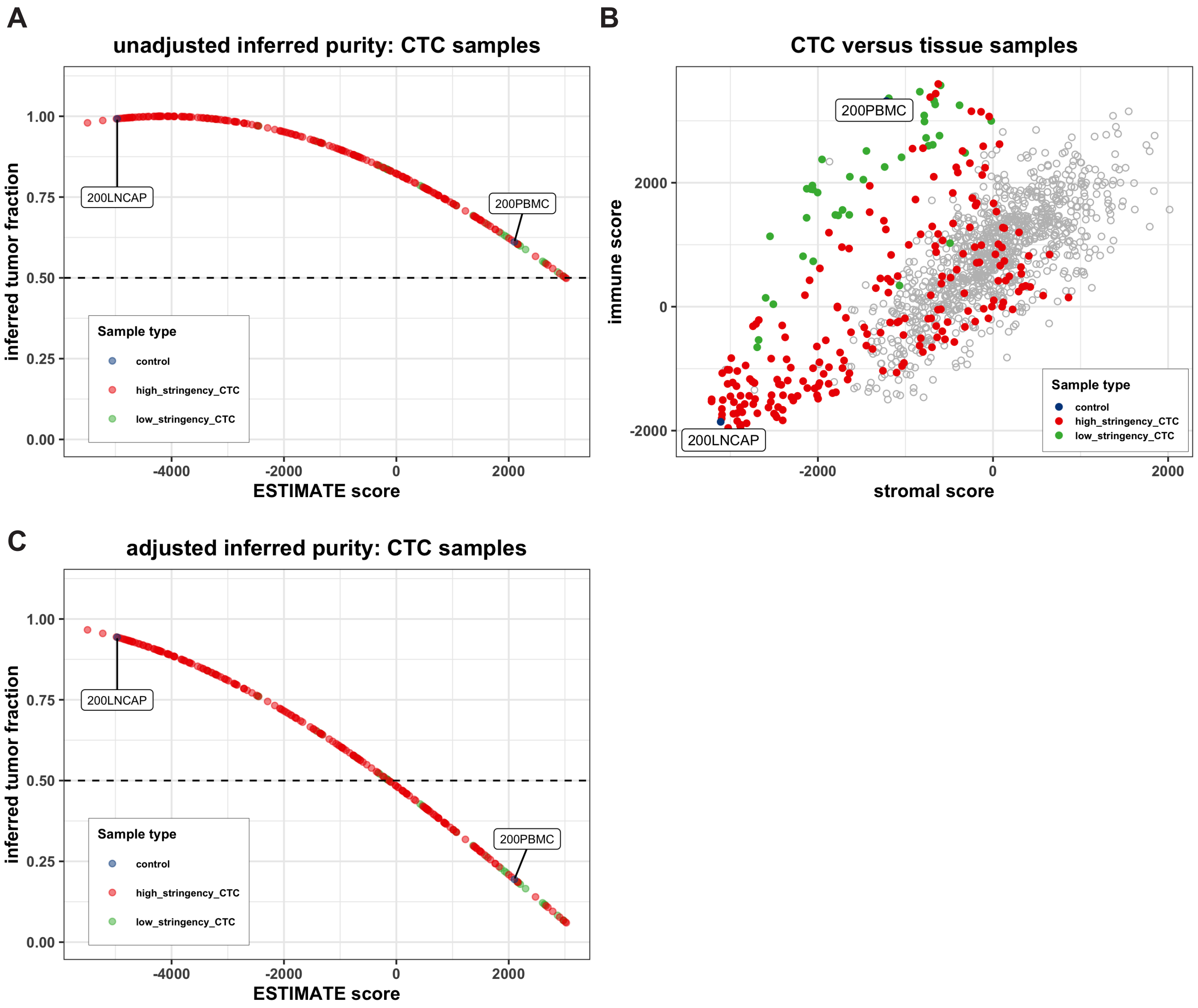

Figure S1. Calibration of ESTIMATE tumor content prediction. (A) ESTIMATE score versus inferred tumor fraction for low stringency (n=41) and high stringency (n=211) CTC samples as well as LNCaP prostate cancer cell line and PBMC controls using the original ESTIMATE equation calibrated for tissue samples. **(B)** ESTIMATE immune versus stromal scores for the CTC and control samples from (A) demonstrating higher immune scores and lower stromal scores than tumor tissue biopsies (n=995) from the original ESTIMATE publication (Yoshihara et al 2013). (grey)**. (C)** ESTIMATE score versus adjusted inferred tumor fraction for CTC samples from (A) after recalibration of the ESTIMATE equation utilizing prostate cell line and PBMC control samples.
