# Supplementary material for: High-Purity CTC RNA Sequencing Identifies Prostate Cancer Lineage Phenotypes Prognostic for Clinical Outcomes
Source: Cancer Discov. Author manuscript; Available in PMC 2025 May 3. (PMC12046329; doi:10.1158/2159-8290.CD-24-1509)
Supplement: Figure S2 [file NIHMS2074075-supplement-Figure_S2.pdf]

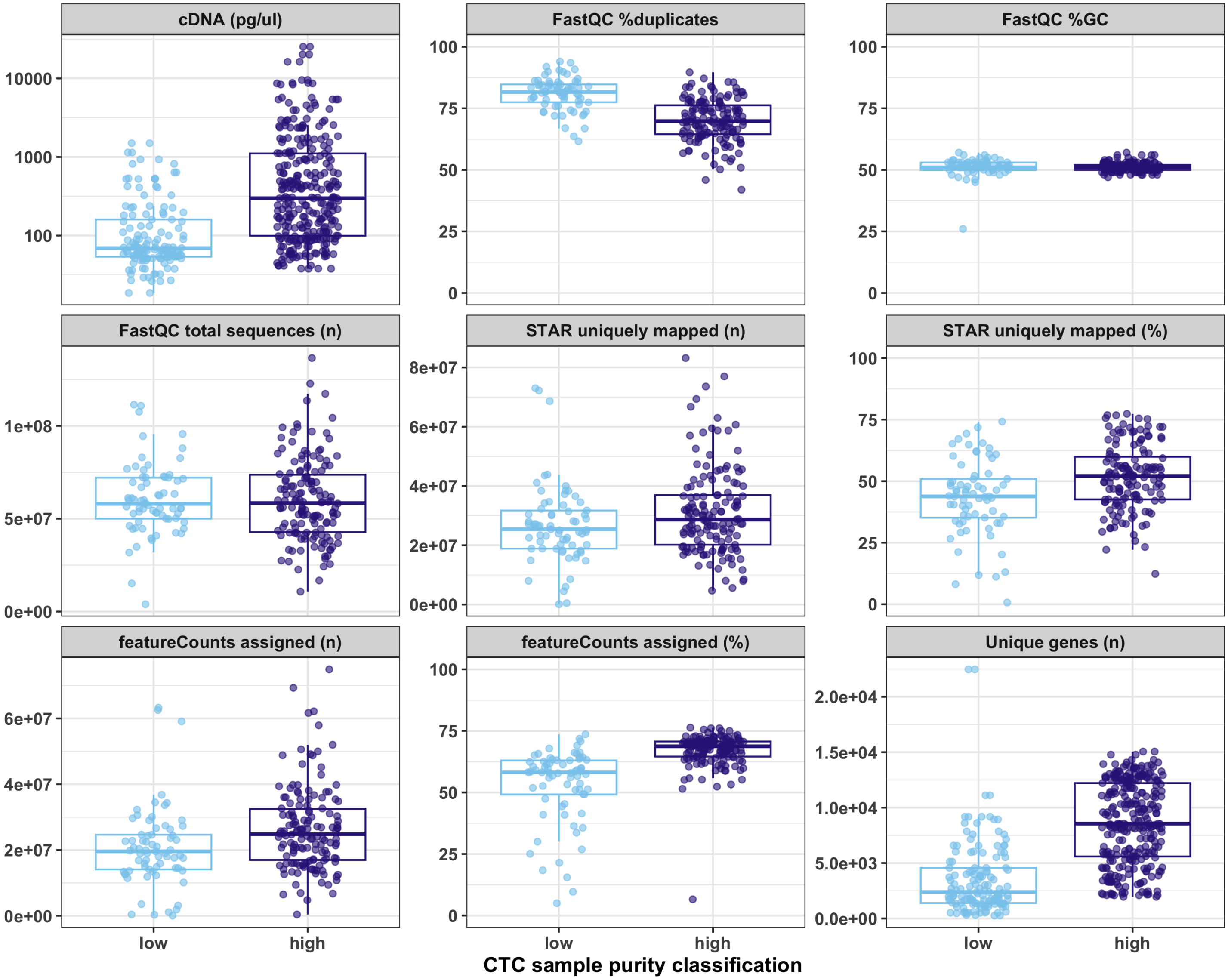

Figure S2. RNA sequencing quality metrics. CTC sample RNA sequencing quality control metrics for low versus high tumor purity CTC samples (high purity defined as tumor fraction ≥ 50% and CibersortX score ≤ 75).
