# Supplementary material for: High-Purity CTC RNA Sequencing Identifies Prostate Cancer Lineage Phenotypes Prognostic for Clinical Outcomes
Source: Cancer Discov. Author manuscript; Available in PMC 2025 May 3. (PMC12046329; doi:10.1158/2159-8290.CD-24-1509)
Supplement: Figure S3 [file NIHMS2074075-supplement-Figure_S3.pdf]

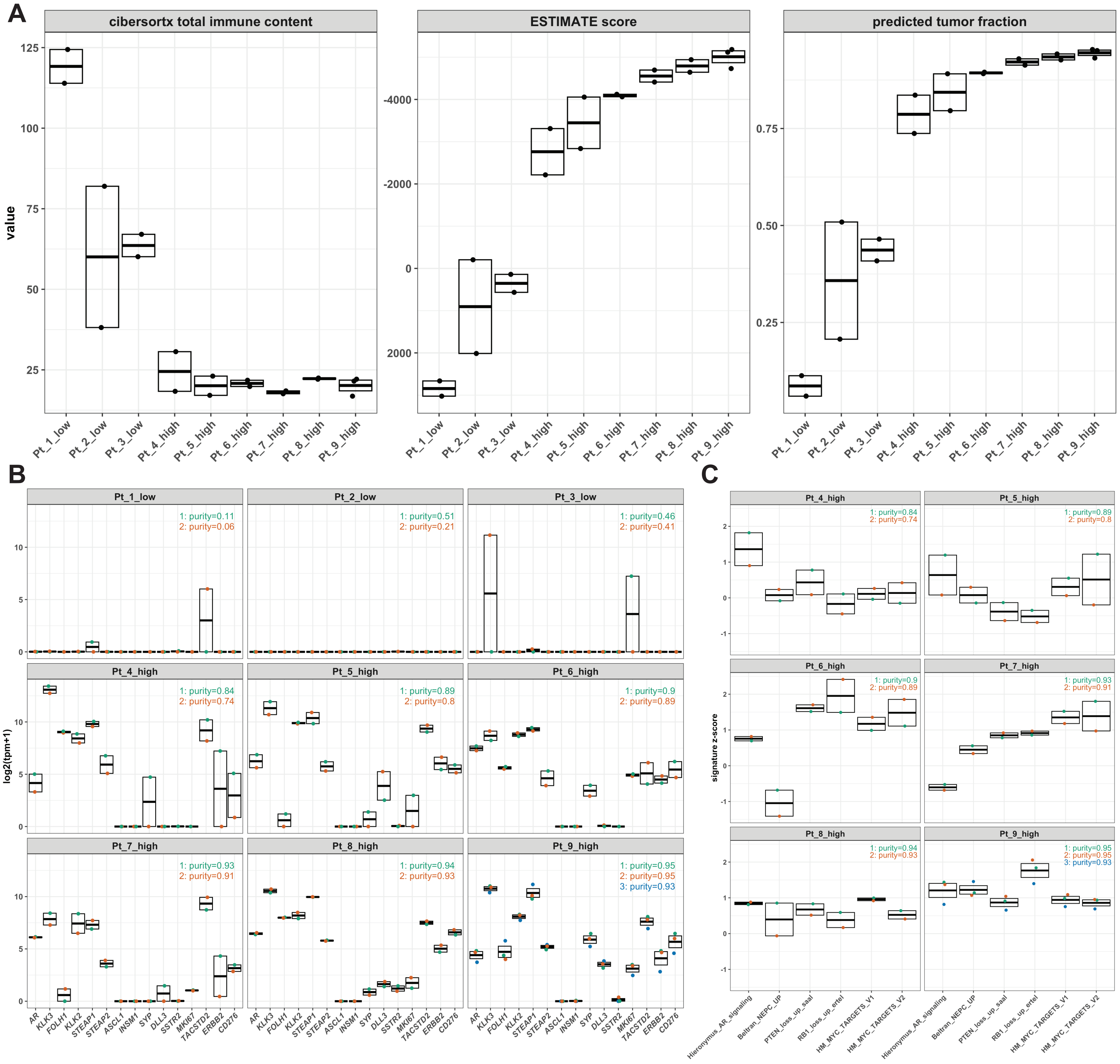

Figure S3. Reproducibility of tumor/immune content assessment, gene expression and pathway scores. (A) cibersortx immune content, estimate score and predicted tumor fraction and **(B)** individual gene expression in technical duplicate CTC samples from 9 patients in the cohort, including three sets of low purity samples (tumor fraction < 50% or CibersortX score > 75) and six sets of high purity samples (tumor fraction ≥ 50% and CibersortX score ≤ 75). **(C)** Pathway signature scores from the 6 patients with high purity technical duplicate samples. Crossbars represent mean +- standard error.
