# Supplementary material for: High-Purity CTC RNA Sequencing Identifies Prostate Cancer Lineage Phenotypes Prognostic for Clinical Outcomes
Source: Cancer Discov. Author manuscript; Available in PMC 2025 May 3. (PMC12046329; doi:10.1158/2159-8290.CD-24-1509)
Supplement: Figure S4 [file NIHMS2074075-supplement-Figure_S4.pdf]

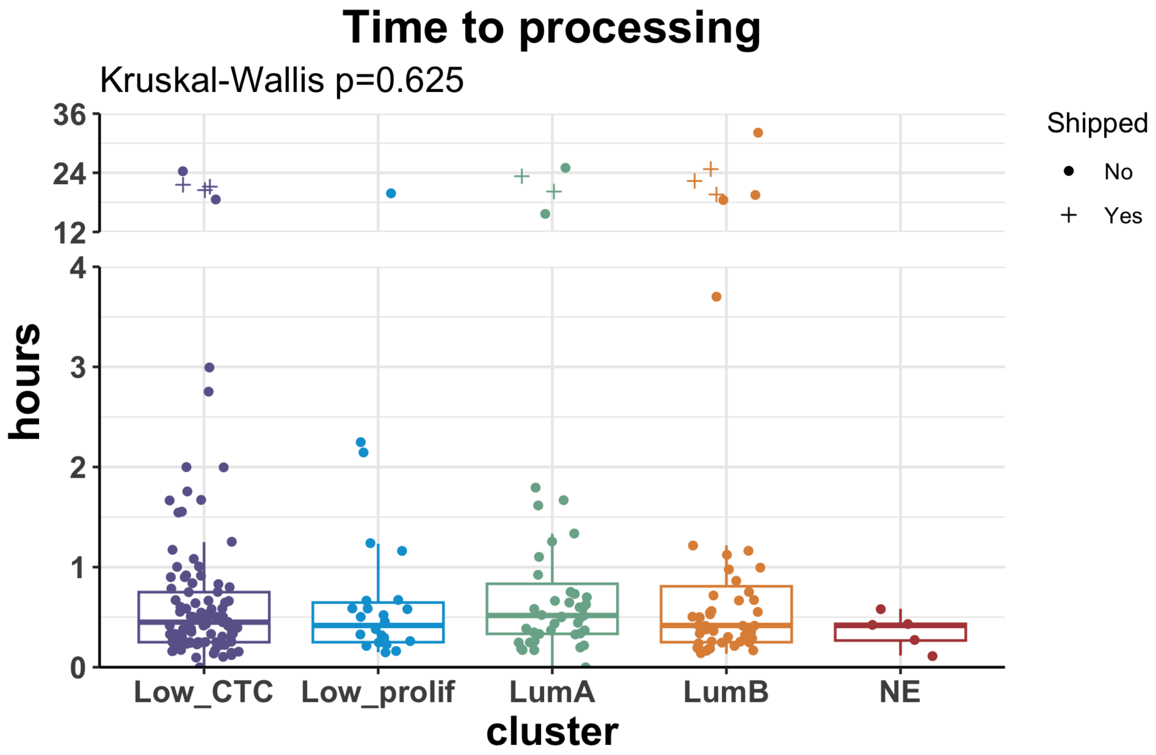

Figure S4. Sample processing is not different between CTC phenotypes. Hours between sample collection and processing as well as shipped versus non-shipped status across CTC phenotype groups. (Low_CTC n=111, Low_prolif n=26, LumA n=43, LumB n=51, NE n=5).
