# Supplementary material for: High-Purity CTC RNA Sequencing Identifies Prostate Cancer Lineage Phenotypes Prognostic for Clinical Outcomes
Source: Cancer Discov. Author manuscript; Available in PMC 2025 May 3. (PMC12046329; doi:10.1158/2159-8290.CD-24-1509)
Supplement: Figure S5 [file NIHMS2074075-supplement-Figure_S5.pdf]

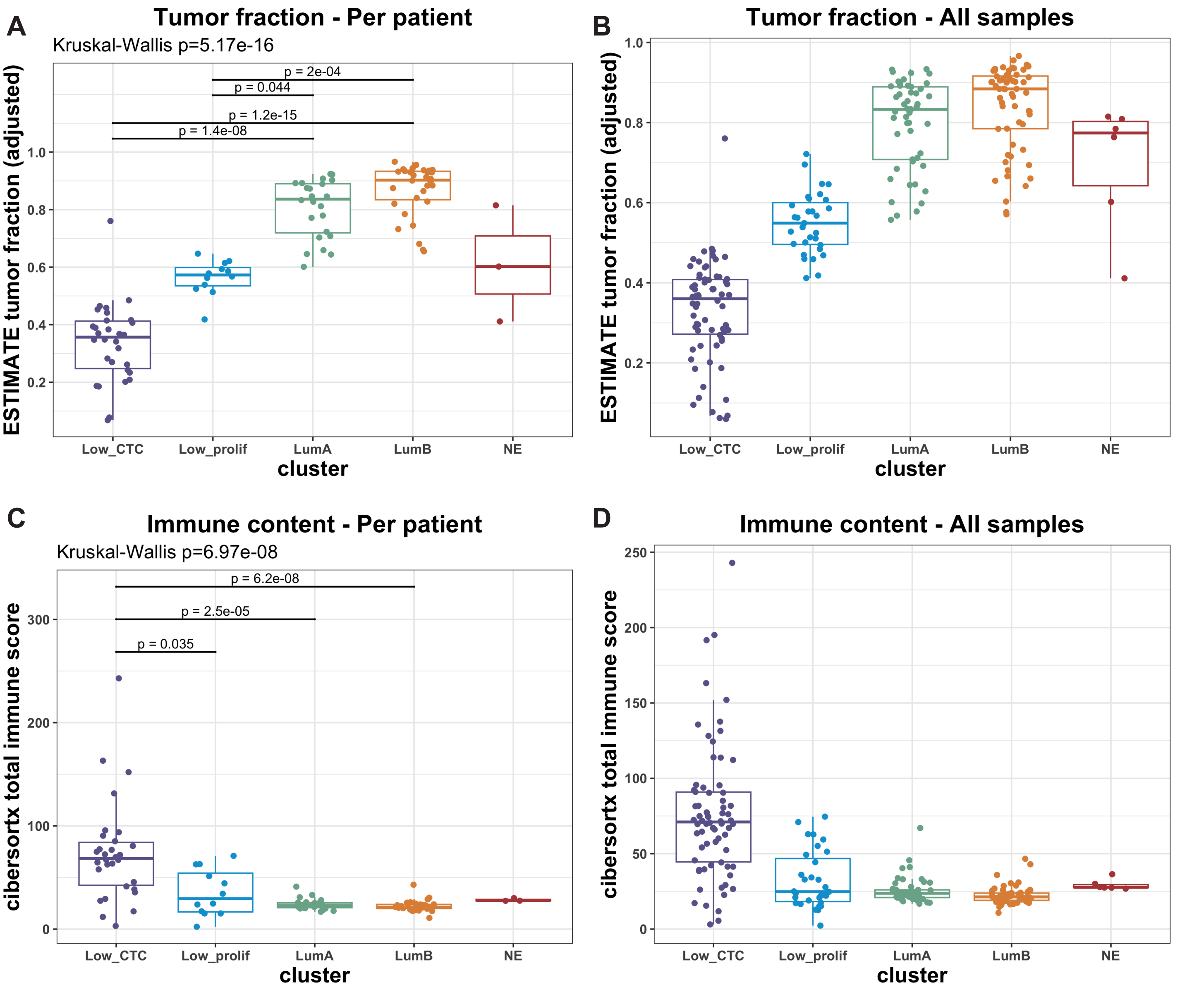

Figure S5. Tumor fraction and immune content across CTC phenotypes. (A) ESTIMATE inferred tumor fraction across CTC phenotypes. For patients with multiple CTC samples, the highest purity sample is included (Low_CTC n=30, Low_prolif n=12, LumA n=24, LumB n=31, NE n=3) **(B)** ESTIMATE inferred tumor fraction for all 210 sequenced samples (Low_CTC n=64, Low_prolif n=31, LumA n=49, LumB n=60, NE n=6). No statistical comparisons are made due to the inclusion of multiple CTC collections for patients who underwent longitudinal sampling. (**C**) CIBERSORTx immune content scores across CTC phenotypes. For patients with multiple CTC samples, the highest purity sample is included (Low_CTC n=30, Low_prolif n=12, LumA n=24, LumB n=31, NE n=3). **(D)** CIBERSORTx immune content scores for all 210 sequenced samples (Low_CTC n=64, Low_prolif n=31, LumA n=49, LumB n=60, NE n=6). No statistical comparisons are made due to the inclusion of multiple CTC collections for patients who underwent longitudinal sampling.
