# Supplementary material for: High-Purity CTC RNA Sequencing Identifies Prostate Cancer Lineage Phenotypes Prognostic for Clinical Outcomes
Source: Cancer Discov. Author manuscript; Available in PMC 2025 May 3. (PMC12046329; doi:10.1158/2159-8290.CD-24-1509)
Supplement: Figure S7 [file NIHMS2074075-supplement-Figure_S7.pdf]

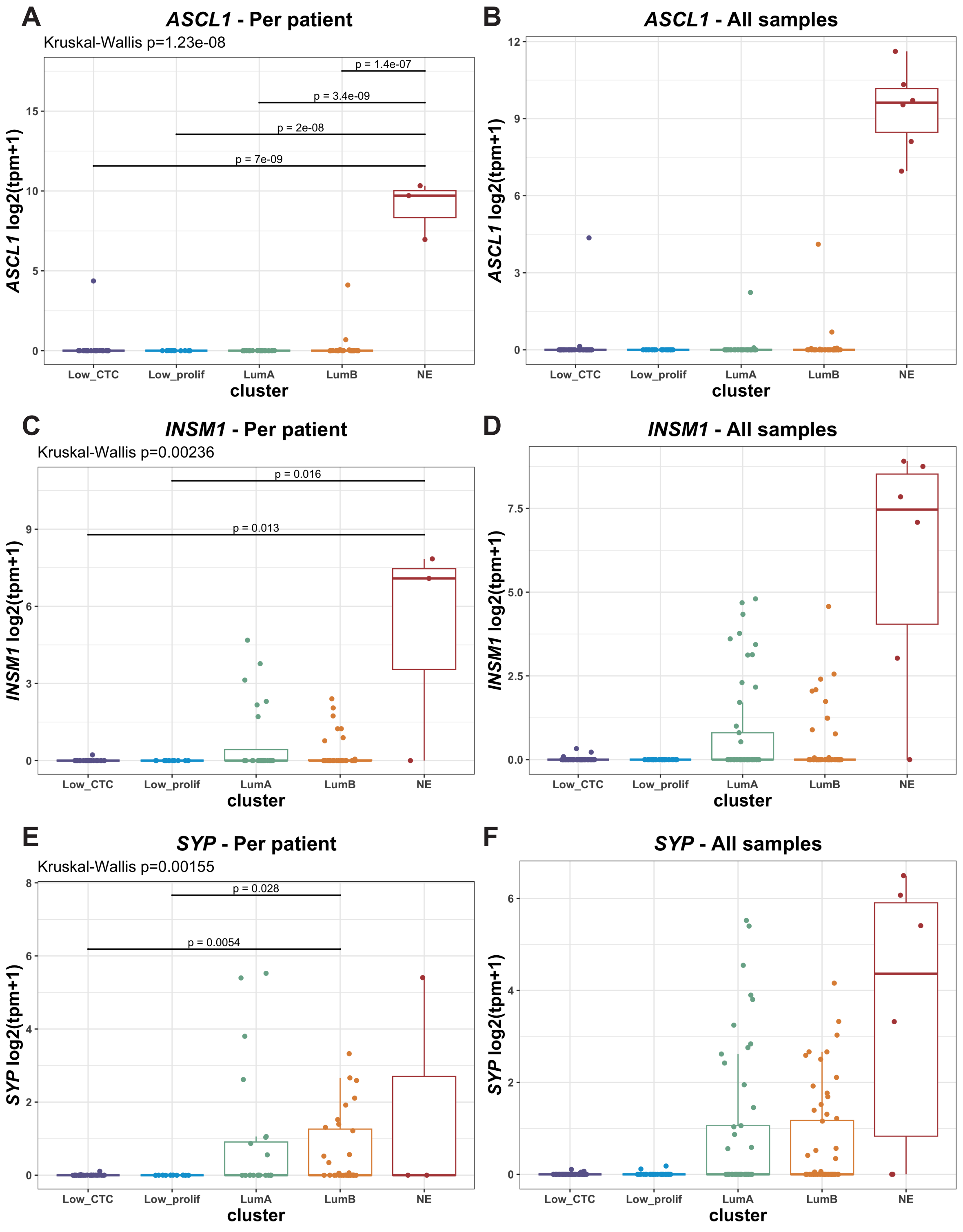

Figure S7. Expression of neuroendocrine genes across CTC phenotypes. Expression of **(A-B)** *ASCL1*, **(C-D)** *INSM1* and **(E-F)** *SYP*. **(A,C,E)** For patients with multiple CTC samples, the highest purity sample is included (Low_CTC n=30, Low_prolif n=12, LumA n=24, LumB n=31, NE n=3). **(B,D,F)** All 210 sequenced samples (Low_CTC n=64, Low_prolif n=31, LumA n=49, LumB n=60, NE n=6). No statistical comparisons are made due to the inclusion of multiple CTC collections for patients who underwent longitudinal sampling.
