# Supplementary material for: High-Purity CTC RNA Sequencing Identifies Prostate Cancer Lineage Phenotypes Prognostic for Clinical Outcomes
Source: Cancer Discov. Author manuscript; Available in PMC 2025 May 3. (PMC12046329; doi:10.1158/2159-8290.CD-24-1509)
Supplement: Figure S8 [file NIHMS2074075-supplement-Figure_S8.pdf]

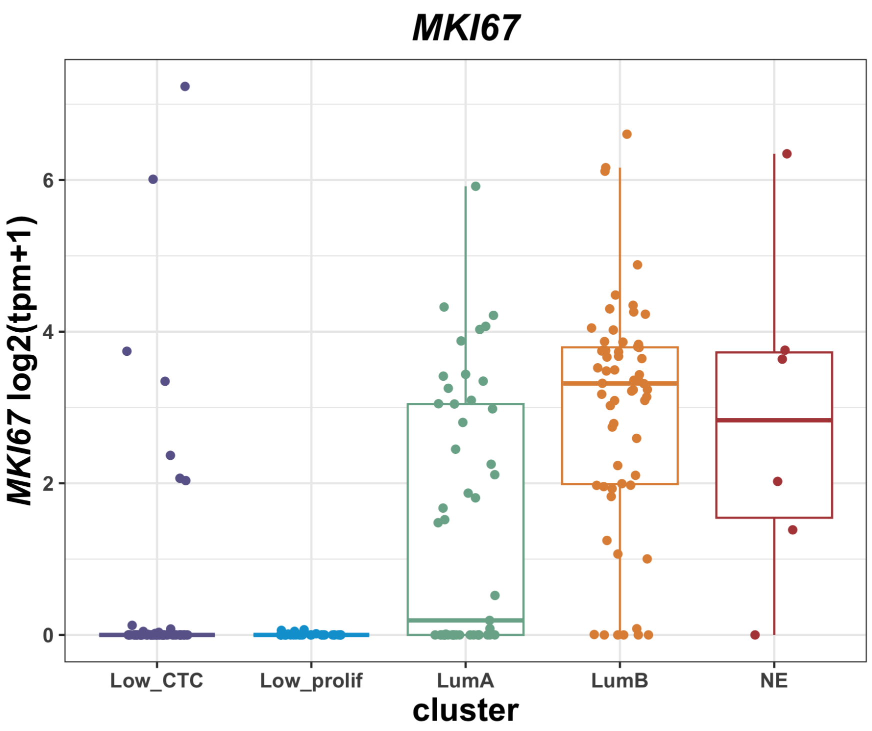

Figure S8. *MKI67* expression in each CTC phenotype cluster for all samples. *MKI67* (Ki-67) gene expression across CTC phenotypes for all 210 sequenced samples (Low_CTC n=64, Low_prolif n=31, LumA n=49, LumB n=60, NE n=6). No statistical comparisons are made due to the inclusion of multiple CTC collections for patients who underwent longitudinal sampling
