# Supplementary material for: High-Purity CTC RNA Sequencing Identifies Prostate Cancer Lineage Phenotypes Prognostic for Clinical Outcomes
Source: Cancer Discov. Author manuscript; Available in PMC 2025 May 3. (PMC12046329; doi:10.1158/2159-8290.CD-24-1509)
Supplement: Figure S9 [file NIHMS2074075-supplement-Figure_S9.pdf]

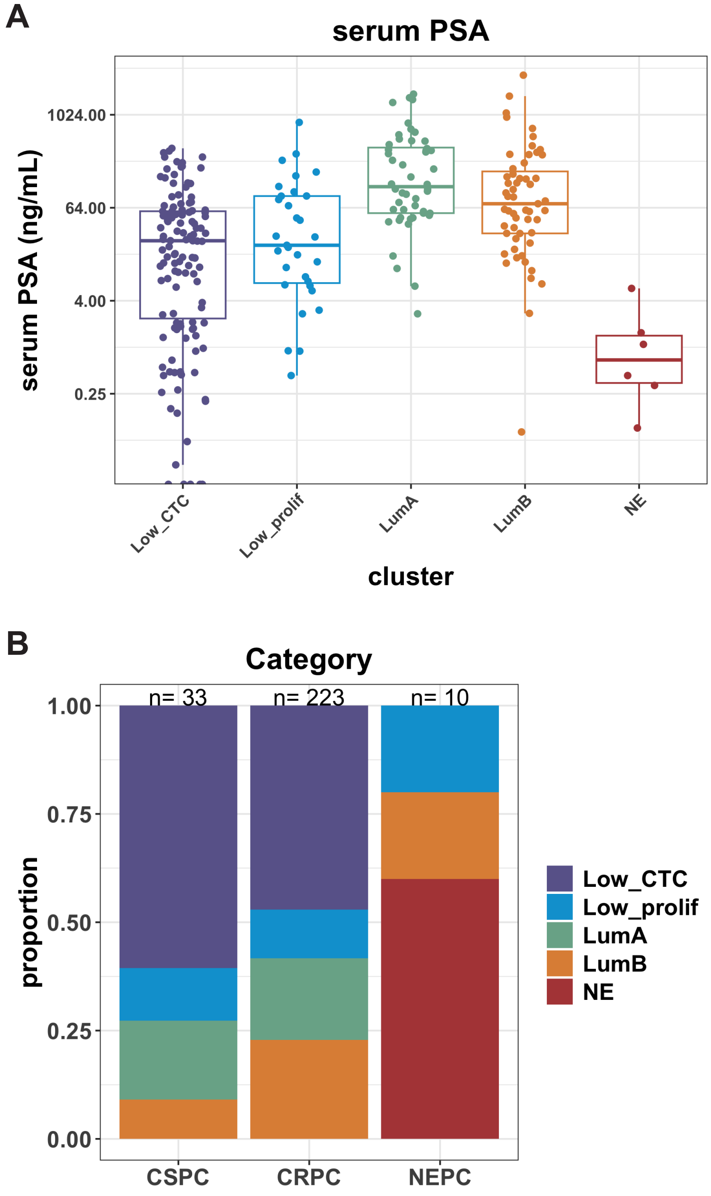

Figure S9. Serum PSA and disease category by CTC phenotype for all samples. (**A**) Serum PSA (ng/ml) at time of CTC collection for all 273 samples (Low_CTC n=129, Low_prolif n=31, LumA n=49, LumB n=60, NE n=6) (**B**) Proportion of samples in each CTC transcriptional phenotype for each disease category (CSPC n=33, CRPC n=220 and NEPC n=10) is shown for all 273 samples. No statistical comparisons are made due to the inclusion of multiple CTC collection timepoints for patients who underwent longitudinal sampling.
