# Supplementary material for: High-Purity CTC RNA Sequencing Identifies Prostate Cancer Lineage Phenotypes Prognostic for Clinical Outcomes
Source: Cancer Discov. Author manuscript; Available in PMC 2025 May 3. (PMC12046329; doi:10.1158/2159-8290.CD-24-1509)
Supplement: Figure S10 [file NIHMS2074075-supplement-Figure_S10.pdf]

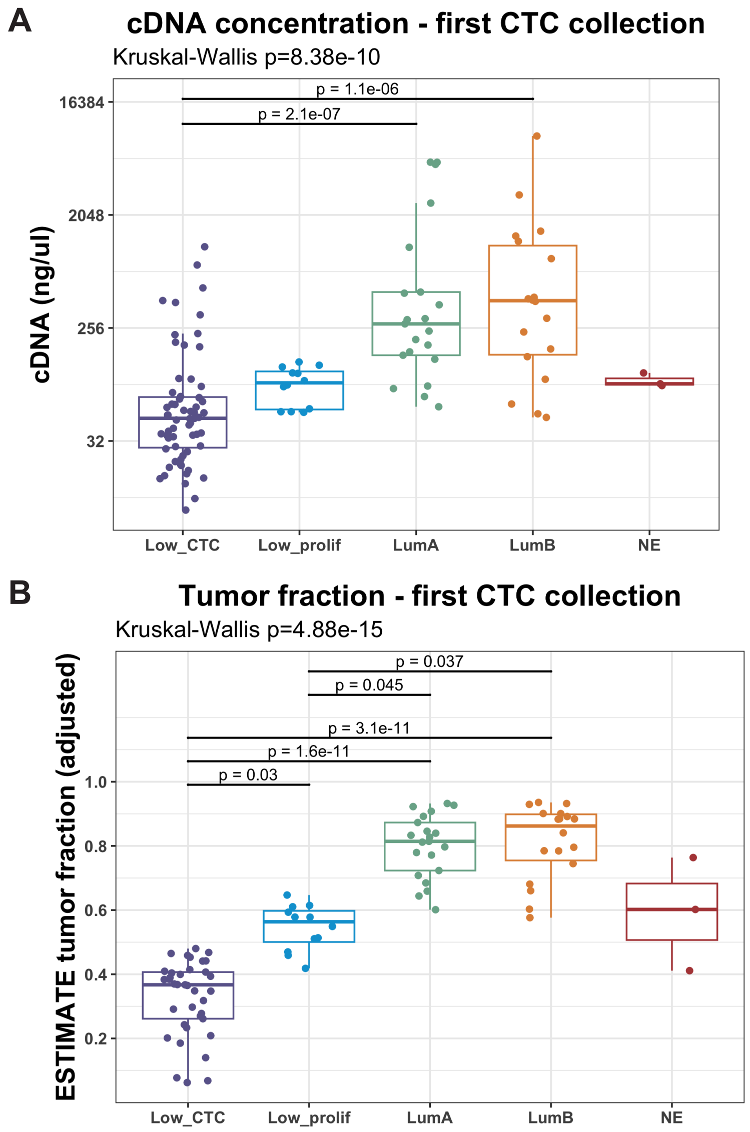

Figure S10. Sample cDNA concentration and tumor fraction by CTC phenotype in the survival analysis subset. (**A**) cDNA concentration as a surrogate for total cellular content and **(B)** ESTIMATE inferred tumor fraction in the survival analysis samples (Low_CTC n=63, Low_prolif n=12, LumA n=21, LumB n=18, NE n=3).
