# Supplementary material for: High-Purity CTC RNA Sequencing Identifies Prostate Cancer Lineage Phenotypes Prognostic for Clinical Outcomes
Source: Cancer Discov. Author manuscript; Available in PMC 2025 May 3. (PMC12046329; doi:10.1158/2159-8290.CD-24-1509)
Supplement: Figure S11 [file NIHMS2074075-supplement-Figure_S11.pdf]

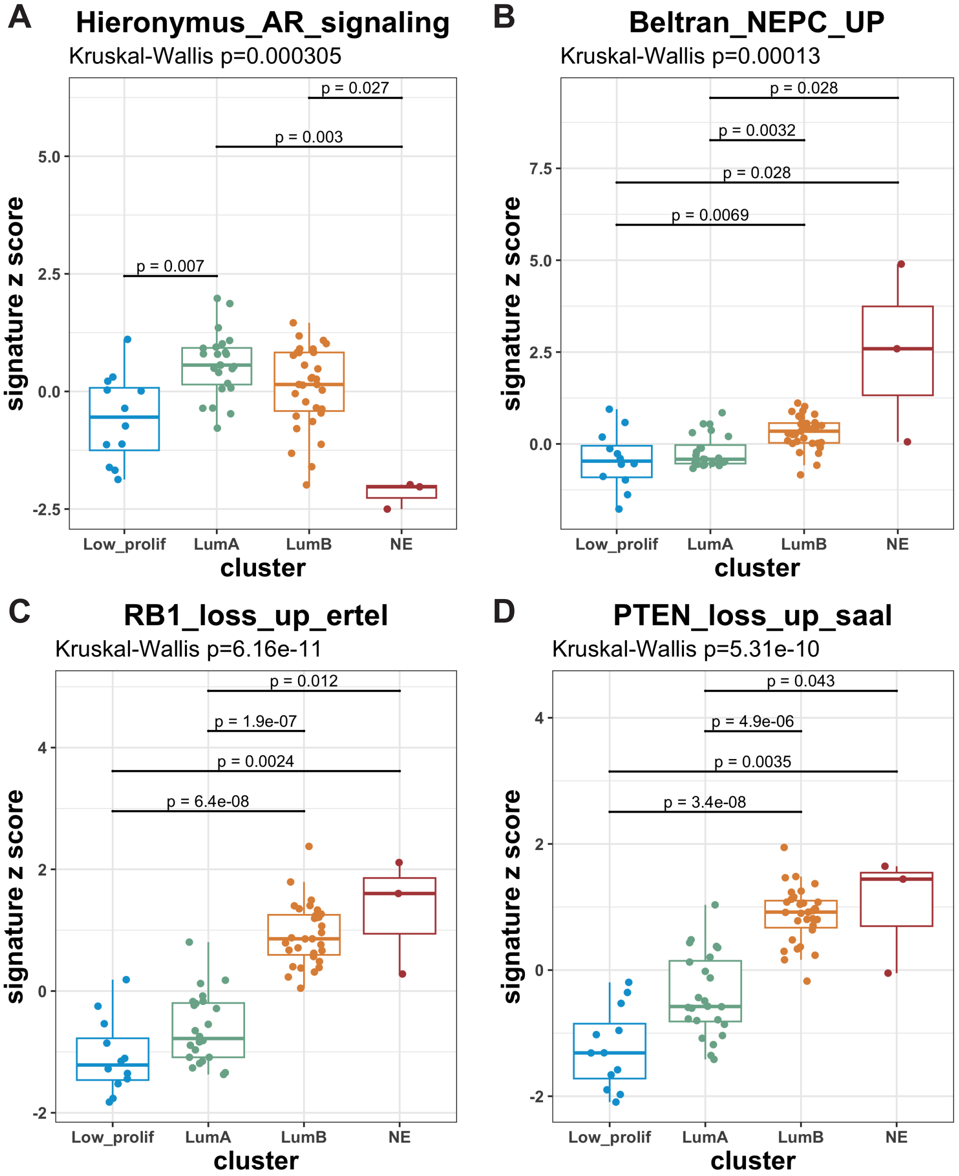

Figure S11. Luminal B phenotype is associated with activation of pathways associated with RB1 and PTEN loss. Only high purity CTC samples were included in pathway analysis; for patients with multiple CTC samples, the highest purity sample is included (Low_prolif n=12, LumA n=24, LumB n=31, NE n=3). **(A-B)** Signature scores across CTC phenotypes for AR and NEPC signature scores held out from k-means clustering. **(C)** RB1 loss signature scores. **(D)** PTEN loss signature scores.
