# Supplementary material for: High-Purity CTC RNA Sequencing Identifies Prostate Cancer Lineage Phenotypes Prognostic for Clinical Outcomes
Source: Cancer Discov. Author manuscript; Available in PMC 2025 May 3. (PMC12046329; doi:10.1158/2159-8290.CD-24-1509)
Supplement: Figure S12 [file NIHMS2074075-supplement-Figure_S12.pdf]

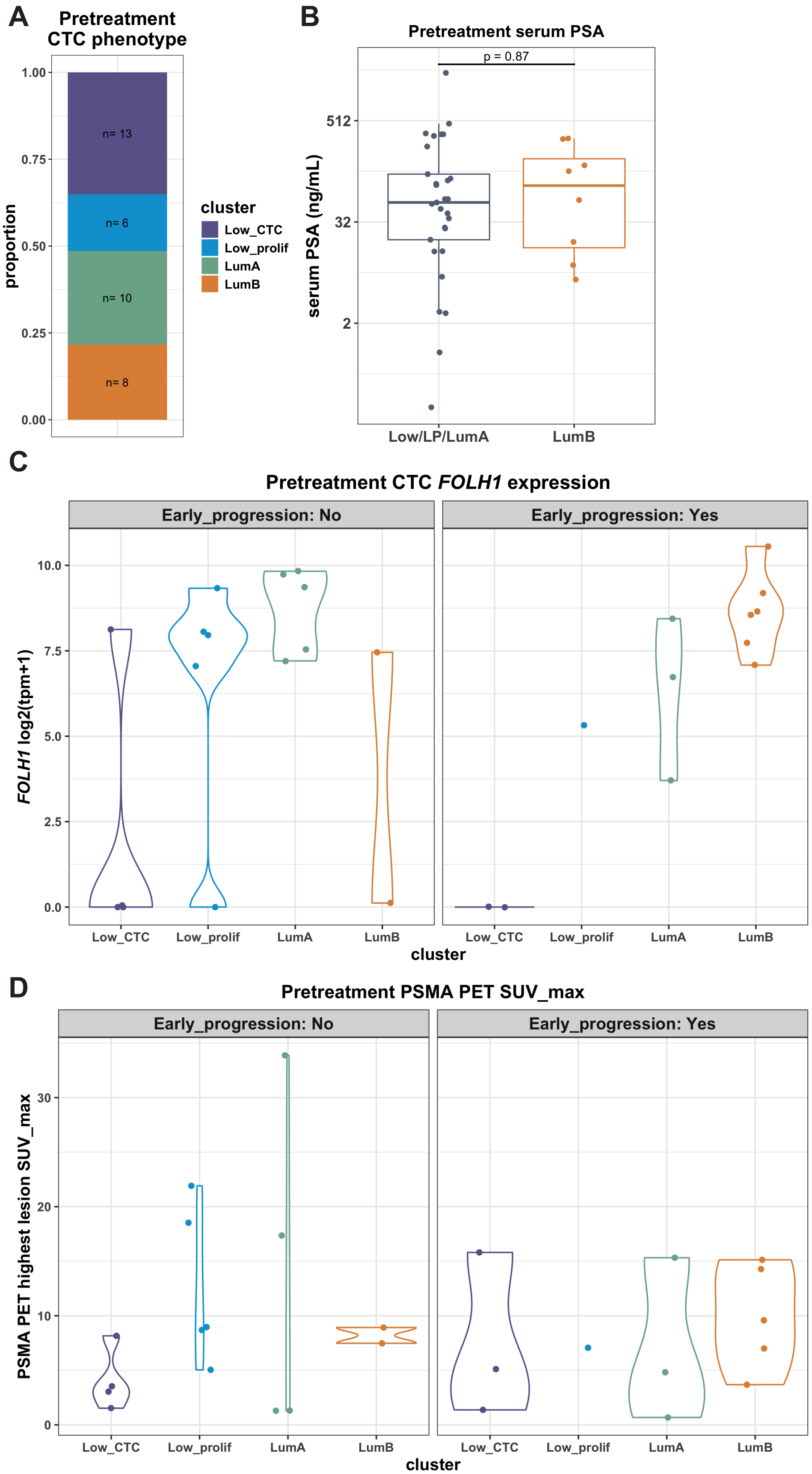


**Figure S12. Pretreatment CTC and PSMA-PET characteristics in the 177Lu-PSMA-617 sub-study cohort. (A)** CTC phenotype of pre-treatment samples in the 177Lu-PSMA-617 sub-study cohort (Low_CTC n=13, Low_prolif n=6, LumA n=10, LumB n=8). **(B)** Pre-treatment serum PSA for patients with LumB (n=8) versus favorable (n=29) CTC phenotypes. **(C-D)** CTC *FOLH1* expression and PSMA-PET highest SUV_max (normalized to physiologic liver SUV_max) by CTC phenotype in pre-treatment samples from patients with and without 177Lu-PSMA-617 early progression (disease progression within the first three cycle of treatment).
