# Supplementary material for: High-Purity CTC RNA Sequencing Identifies Prostate Cancer Lineage Phenotypes Prognostic for Clinical Outcomes
Source: Cancer Discov. Author manuscript; Available in PMC 2025 May 3. (PMC12046329; doi:10.1158/2159-8290.CD-24-1509)
Supplement: Figure S13 [file NIHMS2074075-supplement-Figure_S13.pdf]

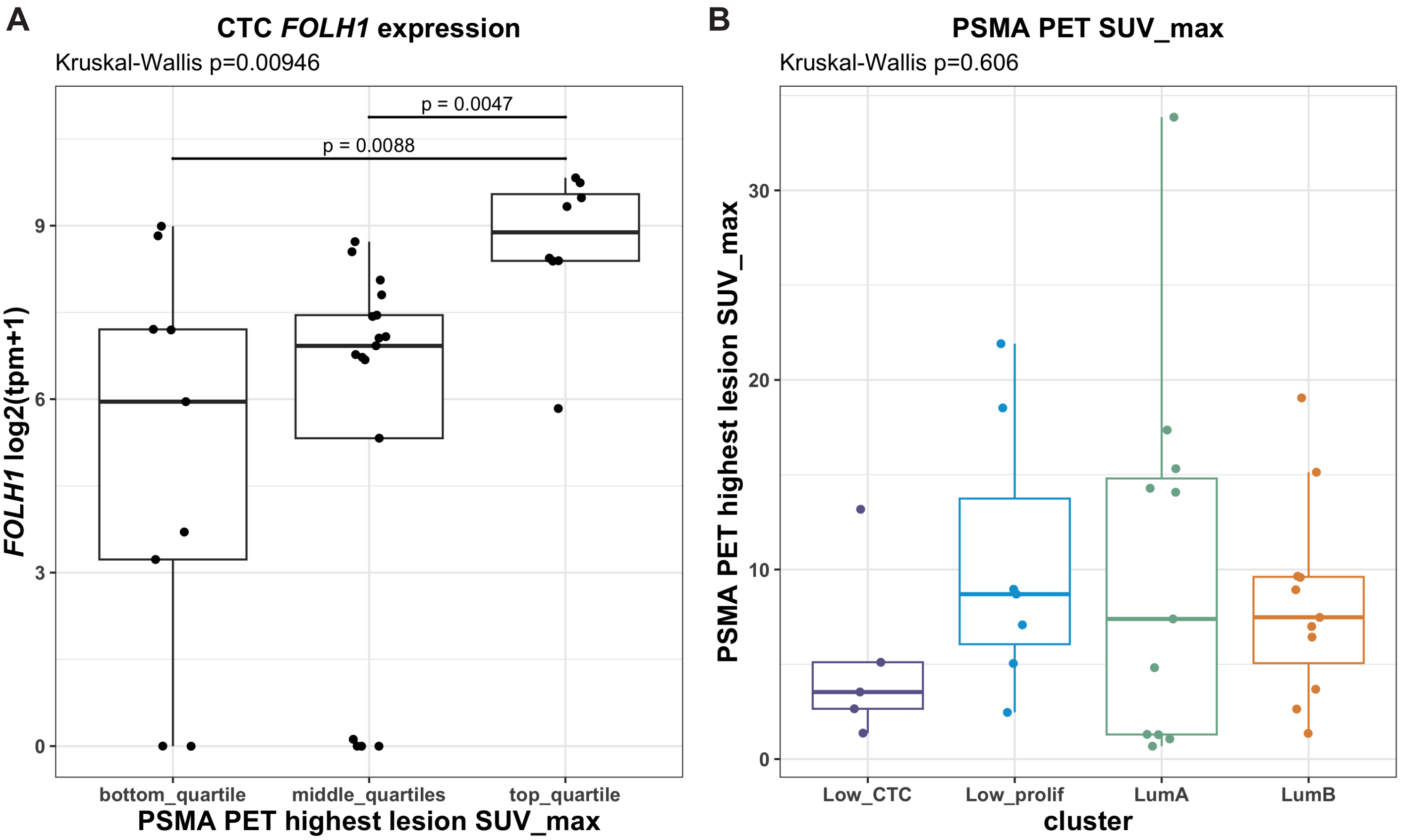

Figure S13. CTC *FOLH1* expression and transcriptional phenotype and PSMA-PET characteristics. (A) CTC FOLH1 expression across PSMA PET highest lesion SUV_max quartiles (bottom_quartile n=9 , middle_quartiles n=17, top_quartile n=8) and **(B)** PSMA PET highest lesion SUV_max across CTC phenotype clusters (Low_CTC n=5, Low_prolif n=7, LumA n=11, LumB n=11) for samples where PSMA PET performed within 18 weeks of CTC sample collection was available. PSMA-PET highest lesion SUV_max was normalized to physiologic liver SUV_max for comparison between studies.
