# Supplementary material for: High-Purity CTC RNA Sequencing Identifies Prostate Cancer Lineage Phenotypes Prognostic for Clinical Outcomes
Source: Cancer Discov. Author manuscript; Available in PMC 2025 May 3. (PMC12046329; doi:10.1158/2159-8290.CD-24-1509)
Supplement: Figure S14 [file NIHMS2074075-supplement-Figure_S14.pdf]

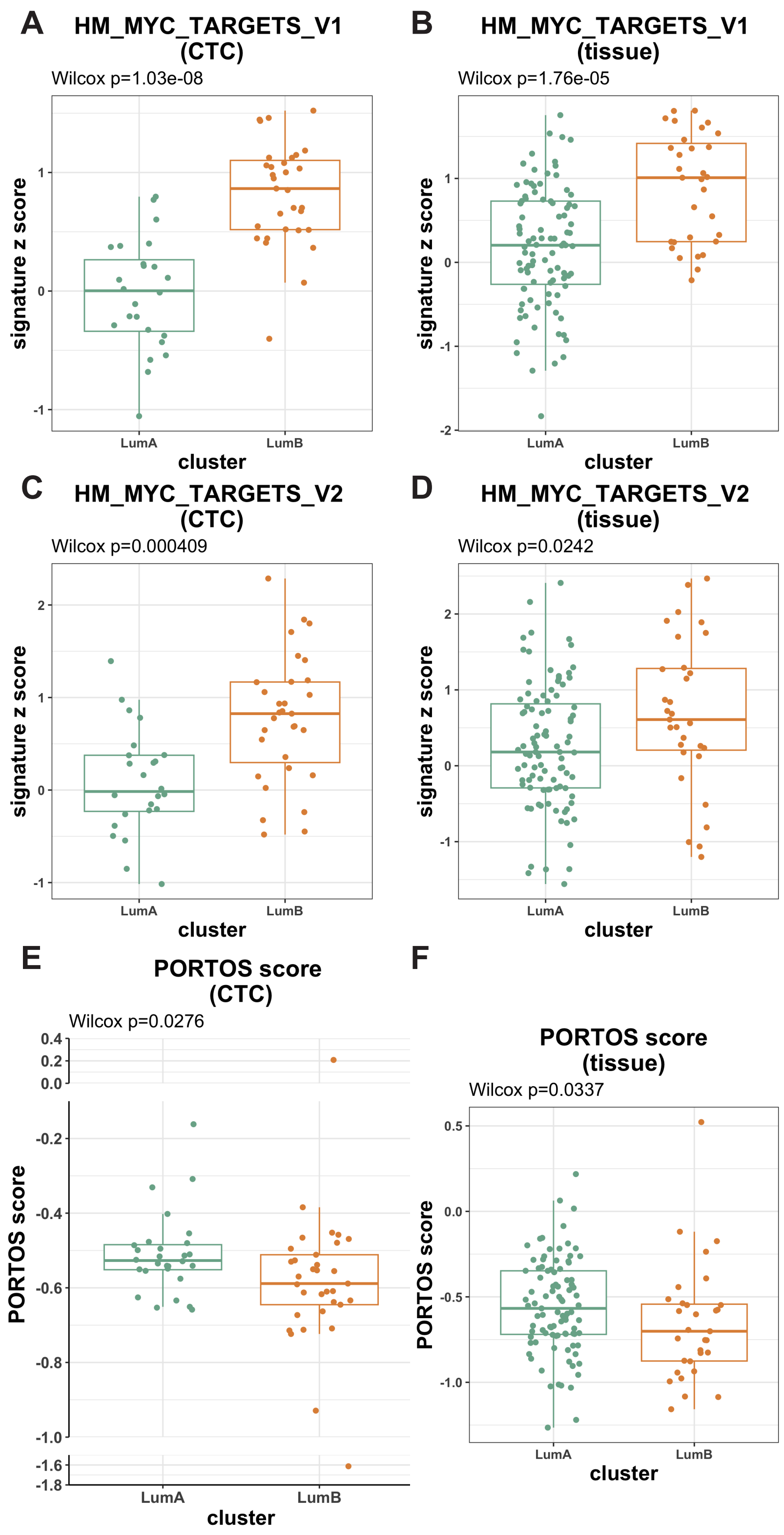

Figure S14. Luminal B phenotype is associated with higher MYC signaling and lower radiation response scores. (A-D) Hallmark MYC signaling signatures are higher in the LumB versus LumA phenotype in both CTC samples (LumA n=24, LumB n=31) and mCRPC tissue biopsies (LumA n=95, LumB n=31). Only high purity CTC samples were included in pathway analysis; patients with multiple CTC samples, the highest purity sample is included. **(E-F)** PORTOS radiation response scores are lower in the LumB versus LumA phenotype in both CTC samples (LumA n=24, LumB n=31) and mCRPC tissue biopsies (LumA n=95, LumB n=31).
