# Supplementary material for: High-Purity CTC RNA Sequencing Identifies Prostate Cancer Lineage Phenotypes Prognostic for Clinical Outcomes
Source: Cancer Discov. Author manuscript; Available in PMC 2025 May 3. (PMC12046329; doi:10.1158/2159-8290.CD-24-1509)
Supplement: Figure S15 [file NIHMS2074075-supplement-Figure_S15.pdf]

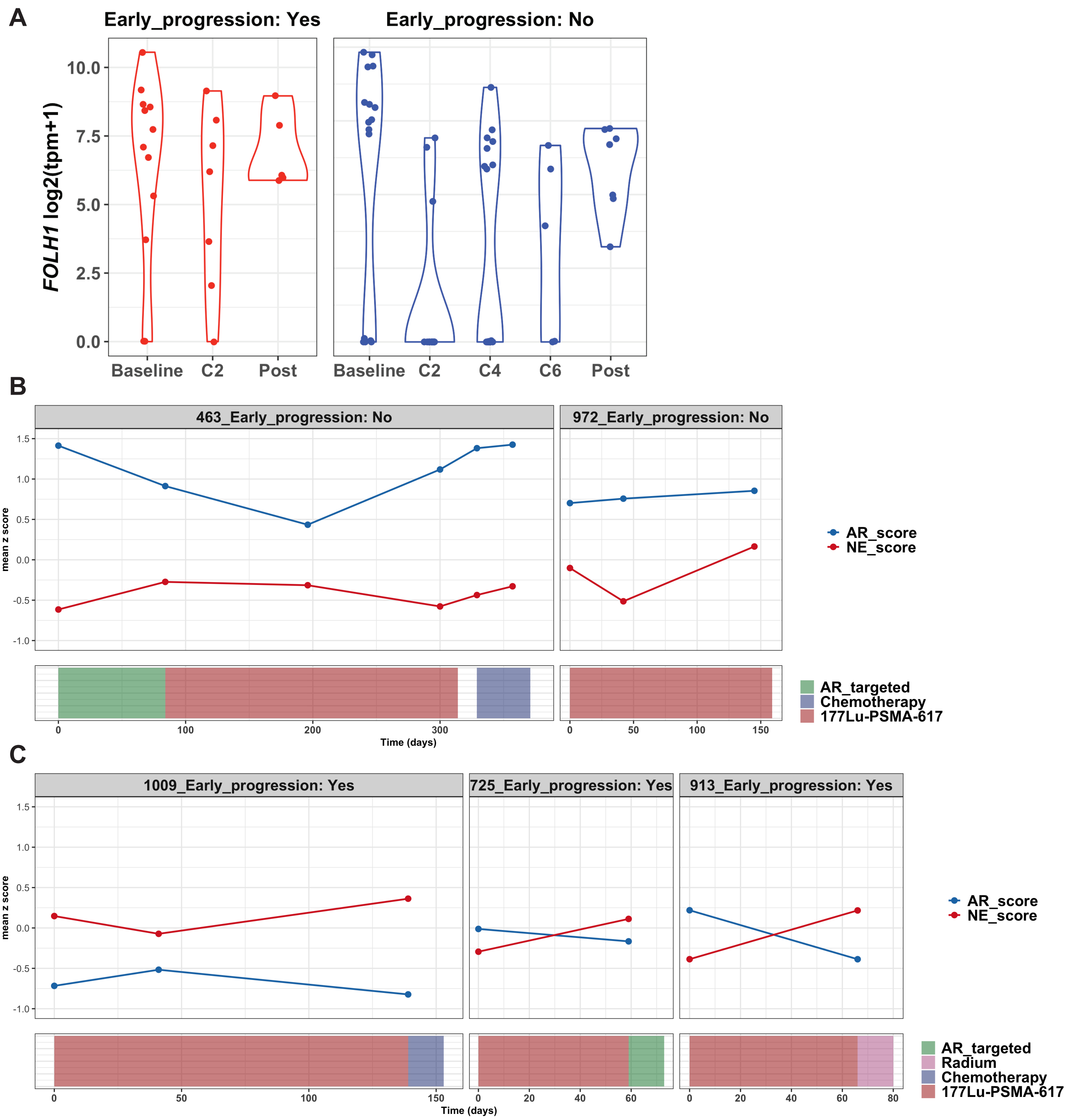

Figure S15. Longitudinal *FOLH1* expression and pathway analysis of CTCs in the 177Lu-PSMA-617 sub-study cohort. (A) CTC *FOLH1* expression of baseline, cycle 2 (C2), cycle 4 (C4), cycle 6 (C6) and progression (Post) samples from patients with and without 177Lu-PSMA-617 early progression (disease progression within the first three cycle of treatment). **(B-C)** Longitudinal evaluation of consensus AR/luminal and neuroendocrine pathway scores during 177Lu-PSMA-617 treatment for **(B)** two patients with stable or increasing AR scores over time who did not have early progression within the first 3 cycles of treatment, in contrast to **(C)** three patients who did have early progression within the first 3 cycles of treatment and showed increased NE scores and decreased AR scores over time.
