# Supplementary material for: High-Purity CTC RNA Sequencing Identifies Prostate Cancer Lineage Phenotypes Prognostic for Clinical Outcomes
Source: Cancer Discov. Author manuscript; Available in PMC 2025 May 3. (PMC12046329; doi:10.1158/2159-8290.CD-24-1509)
Supplement: Figure S16 [file NIHMS2074075-supplement-Figure_S16.pdf]

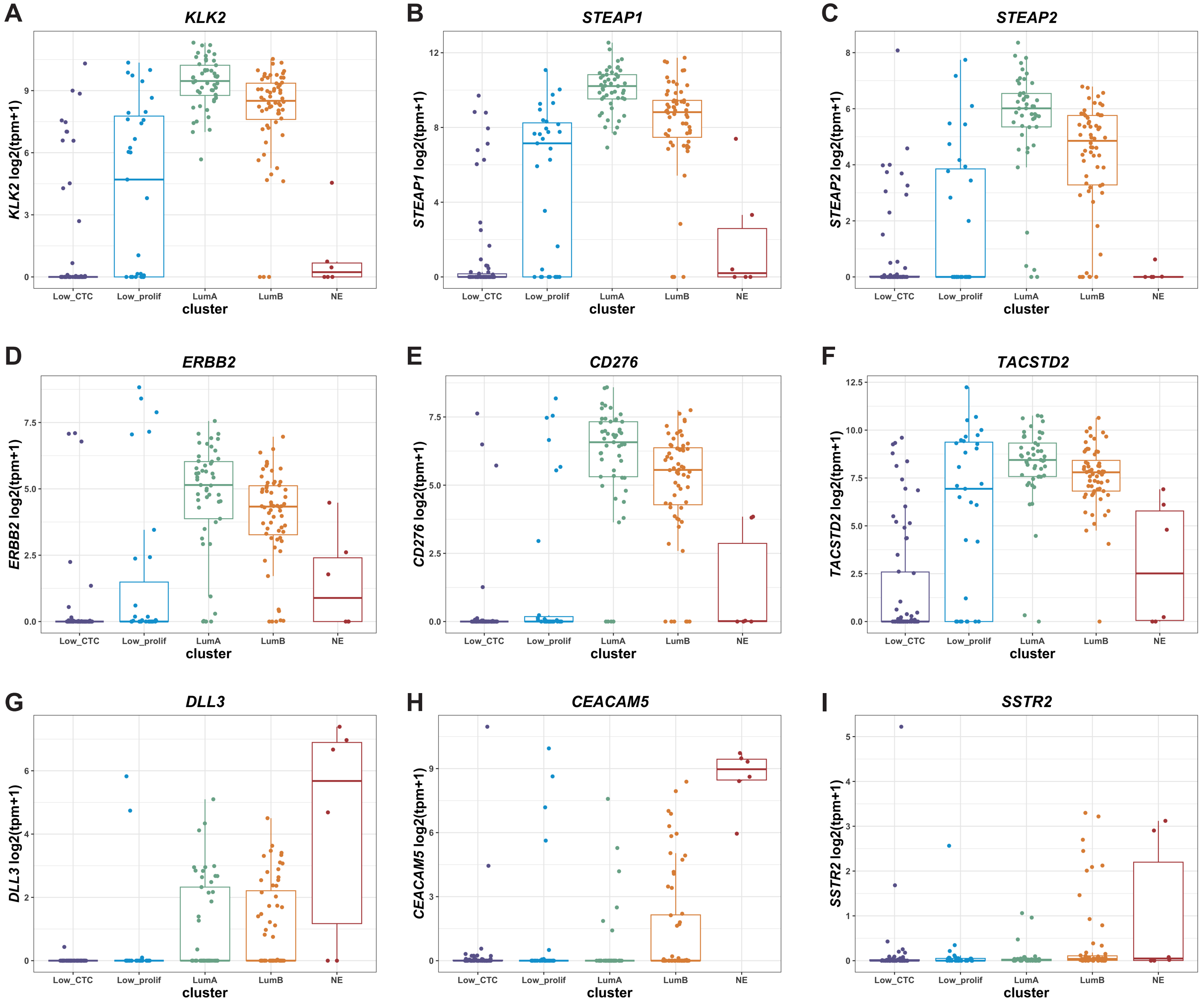

Figure S16. Cell surface target expression across CTC phenotype clusters for all samples. Cell surface target gene expression across CTC phenotypes for all 210 sequenced samples (Low_CTC n=64, Low_prolif n=31, LumA n=49, LumB n=60, NE n=6) for **(A-C)** targets associated with prostate adenocarcinoma (KLK2, STEAP1, STEAP2), **(D-F)** pan-cancer cell surface targets expressed in prostate cancer including TACSTD2 (TROP2), ERBB2 (HER2), and tumor immune checkpoint cell surface protein CD276 (B7H3), and **(G-I)** targets associated with prostate neuroendocrine differentiation (DLL3, CEACAM5, SSTR2). No statistical comparisons are made due to the inclusion of multiple CTC collections for patients who underwent longitudinal sampling.
