# Supplementary material for: High-Purity CTC RNA Sequencing Identifies Prostate Cancer Lineage Phenotypes Prognostic for Clinical Outcomes
Source: Cancer Discov. Author manuscript; Available in PMC 2025 May 3. (PMC12046329; doi:10.1158/2159-8290.CD-24-1509)
Supplement: Figure S17 [file NIHMS2074075-supplement-Figure_S17.pdf]

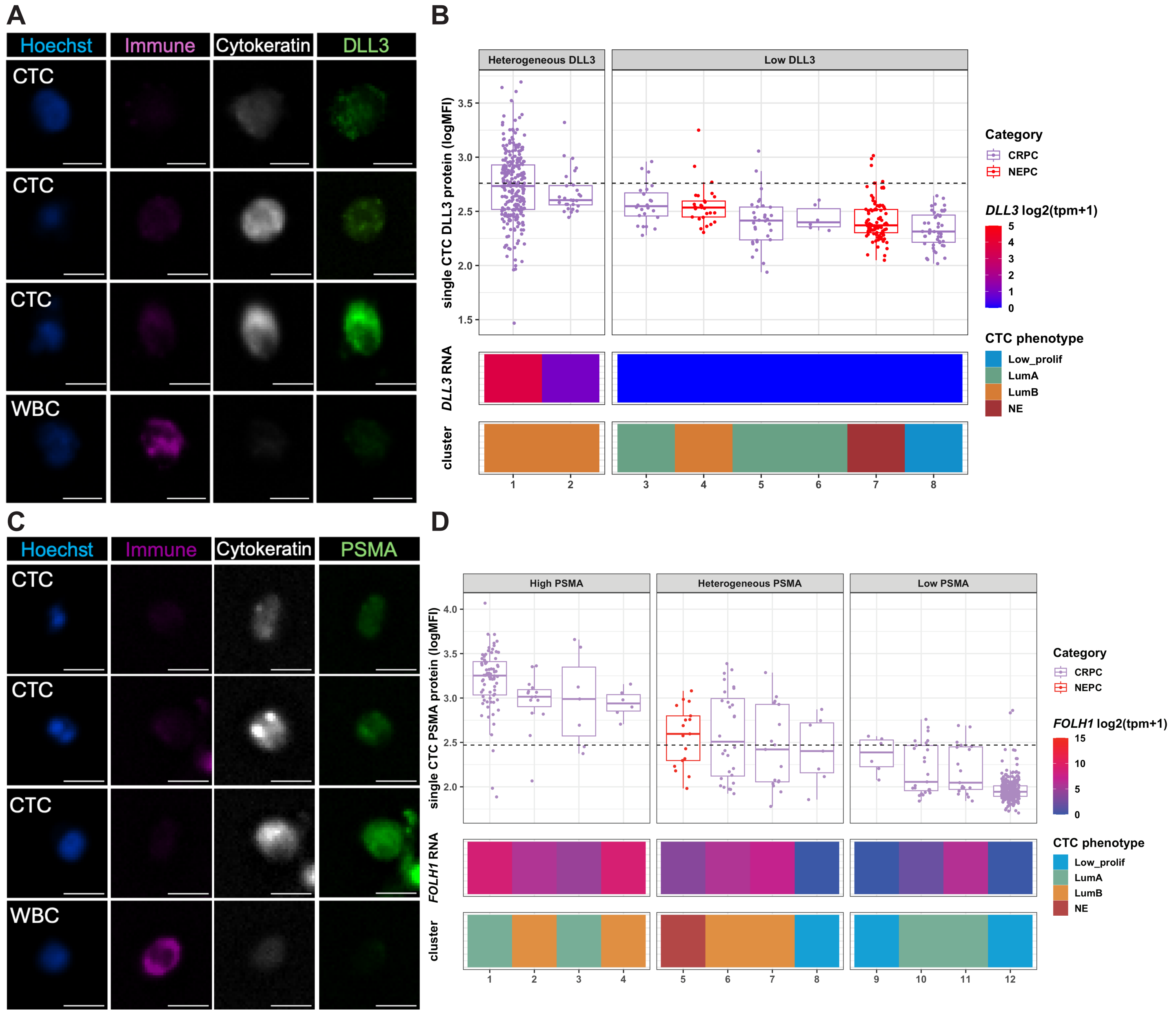

Figure S17. Concordance between protein and RNA expression of cell surface targets in prostate CTCs. (A) representative images and **(B)** quantification of single CTC DLL3 immunofluorescent protein expression demonstrating protein expression heterogeneity by single CTC phenotyping, with concordant detection of DLL3 transcriptional expression in CTC samples with a higher proportion of DLL3-positive CTCs (left). DLL3-positive CTCs are defined as having DLL3 immunofluorescent signal greater than the 95th percentile of non-CTCs (dashed line). **(C)** representative images and **(D)** quantification of single CTC PSMA immunofluorescent protein expression demonstrating samples with high (left), heterogeous (middle) and low (right) PSMA protein expression across CTC transcriptional phenotypes. PSMA-positive CTCs are defined as having PSMA immunofluorescent signal greater than the 95th percentile of non-CTCs (dashed line). Single cell protein expression is quantified as log10 transformed mean fluorescence intensity (logMFI). Scale bar is 10μm.
